# Sustainable and Safe *N*-alkylation of *N*-heterocycles by Propylene Carbonate under Neat Reaction Conditions

**DOI:** 10.3390/ijms25105523

**Published:** 2024-05-18

**Authors:** Andrea Czompa, Dóra Bogdán, Balázs Balogh, Eszter Erdei, Patrik Selymes, Attila Csomos, István M. Mándity

**Affiliations:** 1 Institute of Organic Chemistry, Semmelweis University, Hőgyes Endre utca 7, H-1092 Budapest, Hungary; czompa.andrea@semmelweis.hu (A.C.); bogdan.dora@semmelweis.hu (D.B.); balogh.balazs@semmelweis.hu (B.B.); erdei.eszter@stud.semmelweis.hu (E.E.); patrik.selymes@gmail.com (P.S.); 2 HUN-REN Artificial Transporters Research Group, Institute of Materials and Environmental Chemistry, Research Centre for Natural Sciences Magyar Tudósok körútja 2, H-1117 Budapest, Hungary; 3 Femtonics Ltd., Tüzoltó utca 59, H-1094 Budapest, Hungary; attila.csomos@femtonics.eu; 4 Hevesy György PhD School of Chemistry, Eötvös Loránd University, Pázmány Péter sétány 1/A, H-1117 Budapest, Hungary

**Keywords:** propylene carbonate, *N*-alkylation, heterocycles, neat conditions, green chemistry

## Abstract

A new, eco-friendly process utilising the green solvent propylene carbonate (PC) has been developed to perform *N*-alkylation of *N*-, *O*- and/or *S*-containing heterocyclic compounds. PC in these reactions served as both the reagent and solvent. Importantly, no genotoxic alkyl halides were required. No auxiliary was necessary when using anhydrous PC. Product formation includes nucleophilic substitution with the concomitant loss of water and carbon dioxide. Substrates prepared, including the newly invented PROTAC drugs, are widely used.

## 1. Introduction

Propylene carbonate (PC) has been ranked as one of the greenest solvents according to the GlaxoSmithKline sustainability guide [1]. PC is a carbon-neutral, polar aprotic solvent with a boiling point of 242 °C, serving as an excellent solvent to perform chemical reactions at higher temperatures [2]. All three carbon atoms of the ring are electrophilic sites [3]. PC has been shown to be an efficient solvent for several transformations under various conditions. Various Suzuki–Miyaura reactions were carried out in PC [4]. Interestingly, aside from C–C bond formation, in a few cases, *N*-alkylation occurred and products with a 2-hydroxypropyl side chain were observed, as PC served not only as a reagent, but also as a solvent. Several remarkable examples are reported for alkylation reactions on heteroatoms with PC, including 2-hydroxypropylation of adenine with PC and NaOH in DMF [5]. The *O*-alkylation of phenol was performed in the presence of NaOCH_3_ and with glycerol carbonate as the reagent and solvent [3,6]. The synthesis of *N*-(2,3-dihydroxy)propylanilines and the mechanism of the reaction were also investigated in a reaction of glycerol carbonate with primary amines catalysed by faujasites [7]. Aminolysis as a side reaction was also reported in the transformation of glycerol carbonate with butylamine [8]. The alkylation of resorcinol with ethylene carbonate was carried out using a triorgano-substituted phosphane catalyst [9]. In another study, a similar reaction was executed using alkali-loaded large-pore zeolites as catalysts [10]. Inspired by these results, we have decided to explore the possibilities of applying PC both as a solvent and reagent in order to perform alkylation of certain *N*-, *O*- and *S*-containing substrates in one step. Usually, the alkylation of a nitrogen heteroatom can be achieved with alkyl halides or epoxides [11]; however, these reagents are generally considered as genotoxic agents [12]. To overcome these considerable restrictions, several reactions were carried out where less reactive reagents, like carboxylic acids, were used for *N*-alkylation [13]. However, these reactions needed a special catalyst or special experimental set-up, like the safe handling of hydrogen gas. In a similar way, nitroarenes were utilised for *N*-alkylation under reductive conditions [14]. Alcohols were also used for *N*-alkylation, but this reaction required catalytic conditions as well [15,16]. In 2010, M. Selva and co-workers published the selective synthesis of bis-*N*-(2-hydroxy)alkylanilines with ethylene and propylene carbonate [17], respectively, starting with primary aniline and in the presence of a catalytic amount of phosphonium-based ionic liquids. They conclude that the reactions with PC take place at higher temperatures than those for ethylene carbonate (EC), but only in the presence of phosphonium ionic liquid. By studying the kinetic properties of the reaction of aniline with EC, they found that in the first two hours, the concentration of *N*-2-hydroxyethylaniline increased as the concentration of starting aniline decreased, but in the following hours, the concentration of bis-*N*-(2-hydroxyethyl)aniline increased with a parallel decrease of concentration of the monoalkylated product.

**Scheme 1:**
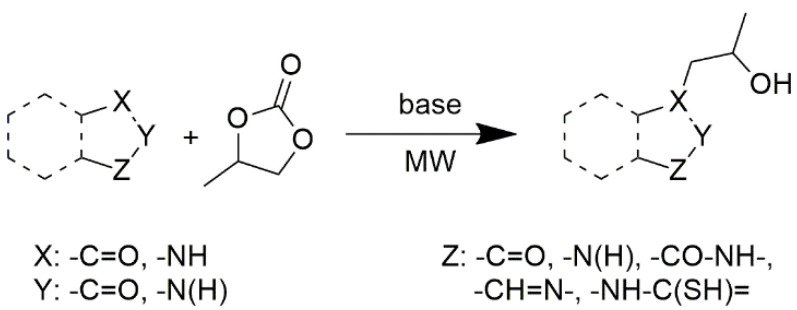
General outline of the performed reactions.

The aim of this work is to study the catalyst-free alkylating nature of PC under alkaline conditions with the aid of a microwave (Scheme 1).

**Figure 1:**
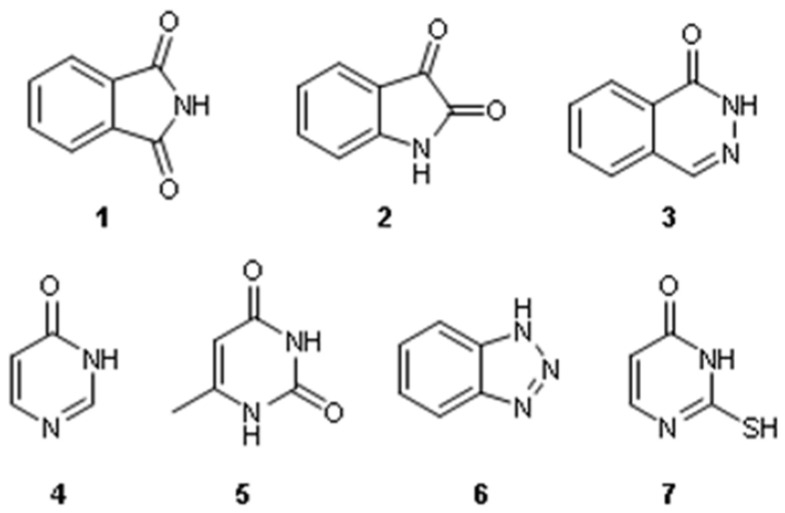
The structures of substrates studied.

Substrates used in the study are phthalimide (**1**), isatin (**2**), phthalazin-1(2*H*)-one (**3**), pyrimidin-4(3*H*)-one (**4**), 6-methylpyrimidine-2,4(1*H*,3*H*)-dione (**5**), 1*H*-benzotriazole (**6**) and 2-thiouracil (**7**) (Figure 1). These compounds have been selected since they are substructures of drug molecules discovered previously. Phthalimide (**1**) is a subunit of anticancer pomalidomide [18] and PROTACs [19], which promote protein degradation [20]. The other investigated heterocycles are structural elements in CNS drugs or antimicrobial agents [21] (isatin), antihistamine azelastine [22] (phthalazinone), antipsychotic risperidone [23] (pyrimidinone), 5-HT2 and α1 receptor antagonist ketanserin [24] (6-methyluracil). 1*H*-benzotriazole derivatives were found to influence metabotropic glutamate receptors [25], while 2-thiouracil is a subunit of the PI3Kδ inhibitor dezapelisib [26].

## 2. Results

Due to our experiences with Suzuki–Miyaura reactions [4a] and green chemical transformation [4b, c, d], we returned to the 1M solution of Na_2_CO_3_ used as a base in the *N*-alkylation reaction of *N*-heterocyclic compounds mentioned before.

All seven substrates contain a nitrogen atom with an acidic hydrogen attached. Our aim was to demonstrate that the corresponding nitrogen nucleophile, formed via proton loss in the presence of a base, can attack the most electrophilic and less sterically hindered side of PC that is the CH_2_ group. In order to achieve our goal, we tried to optimise the quantity of PC. Note, however, that it needs to be used in excess, because it is the reagent and solvent as well.

### 2.1. Phthalimide (***1***)

**Scheme 2:**
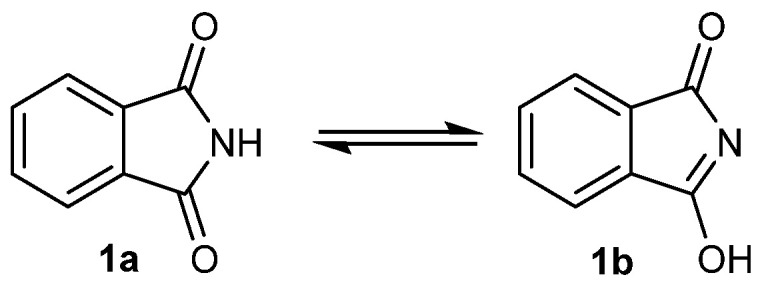
Tautomer of phthalimide.

Our prototype molecule was phthalimide [27,28,29,30,31] with a nitrogen atom in between two electron-withdrawing carbonyl groups. It is a symmetric molecule existing in a tautomeric equilibrium of **1a** and **1b** (Scheme 2).

The reaction mixture was heated either by an oil bath or microwave (MW) irradiation. In order to optimise the amount of substances and reaction conditions, we started using 1 mmol each of the substrates, 1M Na_2_CO_3_ and varied amounts of PC (6, 9, 12 and 18 mmol). The reaction temperature was 130 °C under MW conditions, which corresponds to 150 °C in the oil bath (Table 1). PC in excess amounts was found to be detrimental. In the beginning, we used a temperature of 130 °C in the MW. Unfortunately, our attempt failed with the use of aq. 1M Na_2_CO_3_ (Table 1, entry 1). Similar results were found with 1M of K_2_CO_3_, KOH and NaOAc. Our attempts to add para-toluenesulfonic acid to protonate the hydroxy function and form a better leaving group, as well as raising the temperature to 170 °C (oil bath) and 150 °C (MW), were also unsuccessful.

**Scheme 3:**
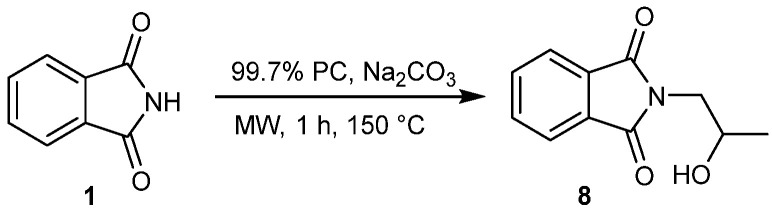
*N*-Hydroxyalkylation of phthalimide (**1**).

It has been suspected that the 99% purity of PC used in excess was a problem. Consequently, a small amount (100 mg) of molecular sieve as a drying agent (Table 1, entry 2) was added. The reaction under these modified conditions resulted in successful *N*-alkylation of phthalimide with a yield of 49% (Scheme 3). Then, the molecular sieve was swapped with anhydrous CaCl_2_ (entry 3) that raised the yield to 63% at 150 °C using the MW, and to 66% at 170 °C in the oil bath, although the reaction time had to be increased from 1 to 4 h (entry 5). Finally, the alkylation with similar ratios of reagents and high-purity (99.7%) PC without a drying agent was carried out; the highest yield of 70% was achieved (150 °C, 1 h, MW; entry 4).

### 2.2. Isatin (***2***)

**Scheme 4:**
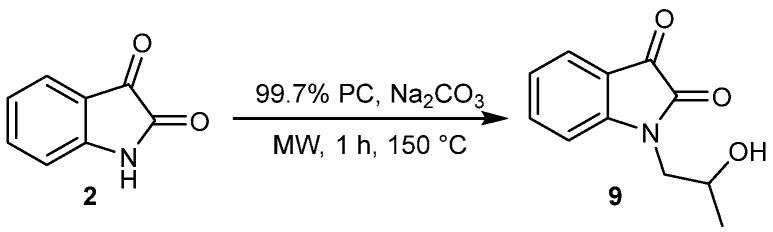
*N*-Alkylation of isatine (**2**).

For this substance (Scheme 4), both conventional heating and microwave irradiation were tested using both 99% and high-purity (99.7%) PC. Unfortunately, at the beginning, the reaction was unsuccessful (see, for example, Table 2, entry 1). In the single successful attempt performed with 99.7% PC, the desired substance [32,33], 1-(2-hydroxypropyl)-1*H*-indole-2,3-dione (**9**), was isolated in a 77% yield (Table 2, entry 2) without a drying agent. We have tried to interpret these experimental results. In a study, isatine was shown to be water sensitive [34,35]; we surmise that a possible ring opening of isatine may contribute to the results found.

### 2.3. Phthalazin-1(2H)-One (***3***)

**Scheme 5:**
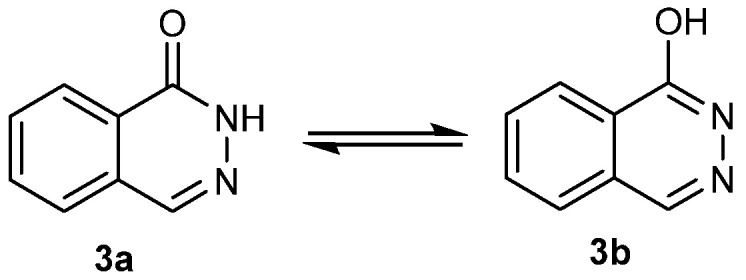
Tautomers of phthalazin-1(2*H*)-one.

Phthalazin-1(2*H*)-one also exists as a tautomeric mixture in which the *exo*-cyclic double bond shifts to the benzocondensed heteroring (Scheme 5) and the hydrogen attached to the nitrogen atom moves to the oxygen atom. In the lactim tautomer we have an aromatic heteroring system, but the hydroxy group formed has lower nucleophilicity; thus, only *N*-alkylation will occur, as in all other cases.

**Scheme 6:**
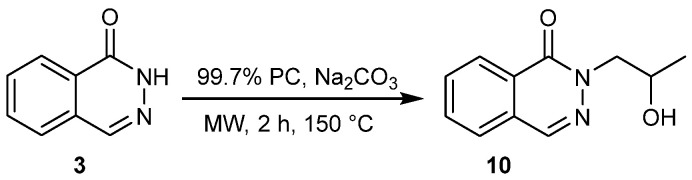
*N*-Alkylation of phthalazin-1(2*H*)-one (**3**).

In the case of phthalazinone, some conclusions can be drawn by comparing reaction methods (oil heating and microwave irradiation) and reaction time. More solvent was used than for the previous cases, because of the increased molecular mass of the substrate. A yield of 28% was found using traditional oil heating in the presence of Na_2_CO_3_ and 1 mmol CaCl_2_ with 99% PC (Table 3, entry 1, Scheme 6). The 4 h MW irradiation improved the yield to 50% (Table 3, entry 2), demonstrating that MW heating is more efficient than oil heating. In a further test, reducing the reaction time to 2 h and utilising 99.7% PC has created the product 2-(2-hydroxypropyl)phthalazin-1(2*H*)-one (**10**) with the highest yield of 55%, even without using the drying agent (Table 3, entry 3). This latter alkylated product is a new compound (Scheme 6). Furthermore, we can start to see a pattern, implying that the absence of water is critical in these *N*-alkylation reactions.

### 2.4. Pyrimidin-4(3H)-One (***4***)

**Scheme 7:**
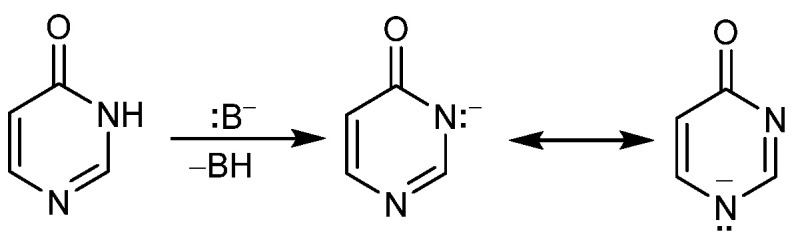
The structure of pyrimidin-4(3*H*)-one anions.

**Scheme 8:**
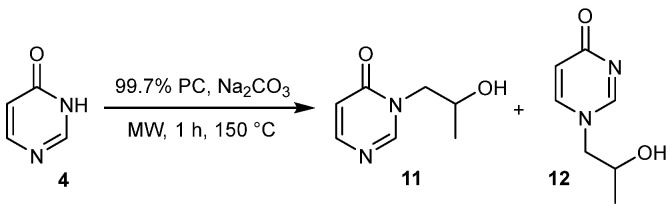
*N*-Alkylation of pyrimidin-4(3*H*)-one (**4**).

In pyrimidin-4(*3H*)-one (**4**), one *N*-atom is in the near proximity of the oxo functional group and the other is further from it, and because of this, it produces two different derivatives, namely a 1*N*- and 3*N*-alkylated products molecule. In addition, the 1*N*-alkylated product (**12**) was the major product (Scheme 7 and Scheme 8).

Oil bath treatment has provided a decent yield of 28% of the 3*N*-alkylated (**11**) product and 54% of the 1*N*-alkylated derivative (**12**) (Table 4, entry 1). Under MW irradiation, 99% purity PC provided a lower amount of product **11** (17%), but **12** was isolated with a similar yield of 58% (data not shown). Testing 99.7% PC resulted in the highest yields of 42% and 57%, respectively, proving the great reactivity of this substance (Table 4, entry 2) towards the alkylating agent. An explanation for this phenomenon could be the higher acidity of the hydrogen attached to the 3*N* due to the proximity of the strong electron-withdrawing carbonyl group. Consequently, the base will abstract this proton and the formed negative charge will be in conjugation with the adjacent double bond, generating a partial negative charge on the 1*N* (Scheme 7). Consequently, it has a higher stability than the other one, because of the resulting quinoidal structure. This accounts for the observed selectivity pattern. Both synthesised products, **11** and **12,** are new compounds.

### 2.5. 6-Methylpyrimidine-2,4(1H,3H)-Dione (***5***)

**Scheme 9:**
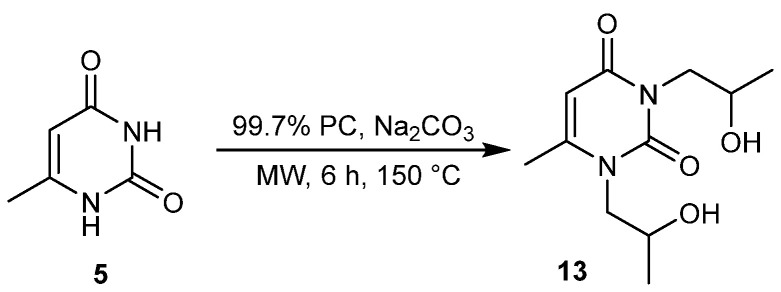
*N*-Alkylation of 6-methylpyrimidine-2,4(1*H*,3*H*)-dione (**5**).

While using solvent in high excess, oil bath conditions required a long time to detect any product at all. Products were barely detected with 99% PC, which made the use of 1 mmol CaCl_2_ necessary (a yield of 6%, Table 5, entry 4). Nevertheless, under MW conditions with less PC, we could synthesise the dialkylated product 1,3-bis(2-hydroxypropyl)-6-methylpyrimidine-2,4(1*H*,3*H*)-dione (**13**) (Scheme 9) with a yield twice as high (Table 5, entry 1).

**Scheme 10:**
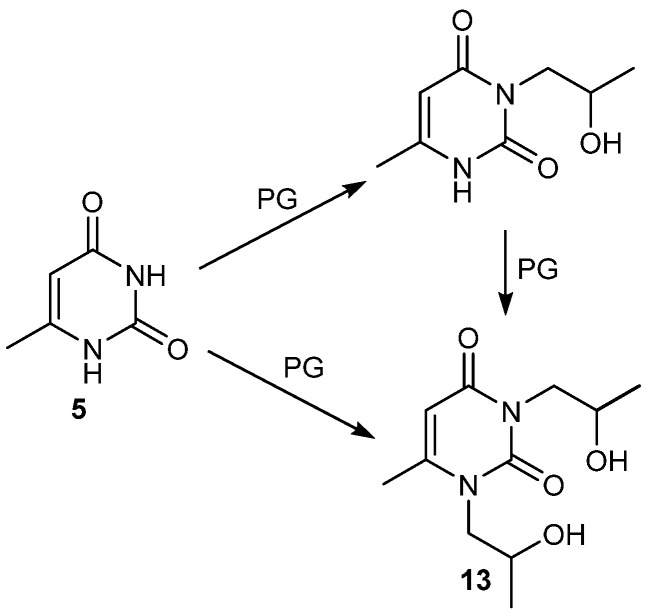
Possible transformation routes in the alkylation of 6-methylpyrimidine-2,4(1*H*,3*H*)-dione.

With higher-quality PC, adding the drying agent was no longer required and we attained a yield of 22% in 2 h (Table 5, entry 2). The highest productivity (49% yield) was achieved by using the MW for 6 h without the drying agent (Table 5, entry 3). The synthesis of 1,3-bis(2-hydroxypropyl)-6-methylpyrimidine-2,4(1*H*,3*H*)-dione (**13**) appeared in a Polish patent synthetised by carcinogenic reagents [36]. In our method, using the green PC as a reagent and solvent allowed us to obtain the dialkylated product (**13**) in a similar yield (49%), with a decreased reaction time of 6 h (Table 5, entry 3). Interestingly, in the transformation of 6-methylpyrimidine-2,4(1*H*,3*H*)-dione (**5**) we could not detect any monoalkylated molecule; only dialkylated compound **13** was formed (Scheme 9). Presumably, the nitrogen atom between the carbonyl groups is alkylated first, leading to a product with a single 2-hydroxypropyl side chain. The latter then undergoes a second reaction, resulting in product **13** (Scheme 10).

It seems that PC is a good alkylating agent, and the proximal electron-donating methyl group does not influence the alkylation of both nitrogen atoms. In order to confirm the alkylating power of the present system, we decided to probe its reactivity with a substrate which does not contain such a strong electron-withdrawing group as the carbonyl group.

### 2.6. 1H-Benzotriazole (***6***)

**Scheme 11:**
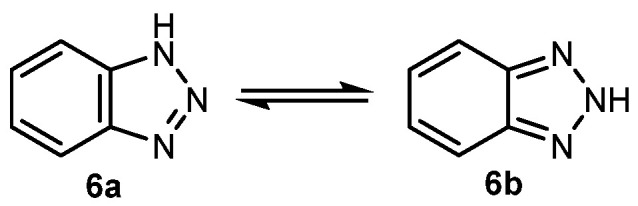
Isomeric 1*H*- and 2*H*-benzotriazoles.

This substrate has three nitrogen atoms in positions 1, 2 and 3 and it has the following two isomers: 1*H*- and 2*H*-benzotriazole (Scheme 11). The nitrogen atoms have an electron-withdrawing effect and, consequently, the attached hydrogen atoms can be removed by a base, while the nucleophiles obtained can be alkylated by PC. The 2-hydroxypropyl side chain obtained after *N*-alkylation has a steric demand. As a result, it is not expected to deliver a dialkylated product at the benzotriazole ring. Furthermore, if one considers that the nucleophile obtained by deprotonation of the 1*N* atom is more stable than the 2*N* atom, alkylation is expected to take place on the 1*N* atom [37,38]. Nevertheless, we were curious to know if *N*-alkylation occurs at all.

**Scheme 12:**
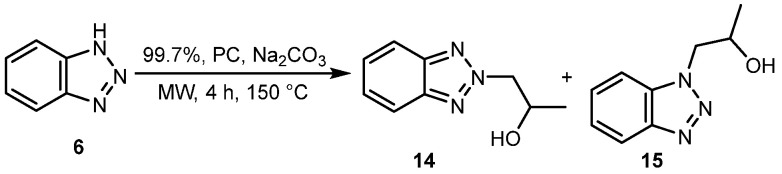
Results with benzotriazole (**6**).

The results of our experiments with benzotriazole are summarised in Table 6. These data show that *N*-alkylation can take place on both nitrogen atoms (Scheme 12). In each case, the quantity of the 1*N*-alkylated product (**15**) was higher than that of the 2*N*-alkylated product (**14**). Using PC in the presence of the drying agent and oil bath heating, we measured 22% of 1-(2*H*-benzotriazol-2-yl)propan-2-ol (**14**) and 47% of 1-(1*H*-benzotriazol-1-yl)propan-2-ol (**15**). Under identical reaction conditions, using microwave irradiation, the amounts of both **14** and **15** increased (entry 2). As usual, the best results were achieved without the drying agent (CaCl_2_) and by utilising high-purity PC under MW conditions (entry 3). As expected, in all cases, the 1*N*-alkylation reaction (product **15**) occurs at a higher percentage (47–55%) than the 2*N*-alkylation process (product **14**, 22–35%).

### 2.7. 2-Thiouracil (***7***)

**Scheme 13:**
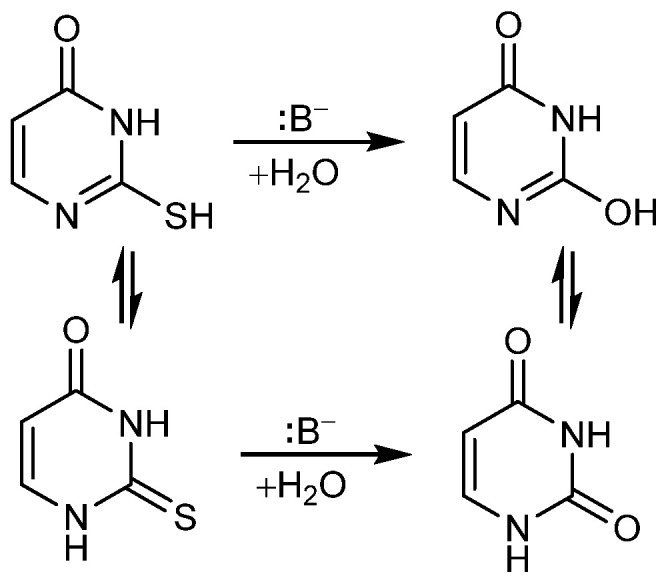
Hydrolysis of 2-thiouracil and its tautomer.

The last substrate in our *N*-alkylation experiments was 2-thiouracil. It is already known that 2-thiouracil and its 5- and 6-methyl derivatives are oxidisable with hypervalent iodine [39], for example, using iodosylbenzene or employing ozone. In each case, the thiocarbonyl moiety is attacked and desulfurisation occurs, creating uracil and carbonyl compounds [40]. According to the literature, 2-thioxo-1,2,3,4-tehtrahydropyrimidin-4-one reacted with propylene oxide in a similar manner [41]. However, propylene oxide is not an environmentally benign reagent. In this report, NaOMe/MeOH, KOH/MeOH and NaOH/H_2_O as base–solvent systems were applied, and in each case, the desired derivatives were isolated in an almost quantitative yield. It seems that, in the presence of a base, the thiol functional group between the two nitrogen atoms of the starting 2-thiouracil is replaced by a hydroxy group, as depicted in Scheme 13.

**Scheme 14:**
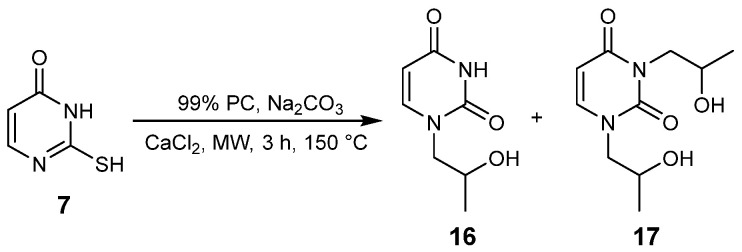
*N*-Alkylation of 2-thiouracil (**7**) with 99% PC and CaCl_2_.

Based on these findings, we were not surprised to obtain two products; both monoalkylated 1-(2-hydroxypropyl) pyrimidine-2,4(1*H*,3*H*)-dione (**16**) and dialkylated-uracil (**17**) were detected (Scheme 14). 1,3-Bis(2-hydroxypropyl)pyrimidine-2,4(1*H*,3*H*)dione (**17**) is a new compound, while 1-(2-hydroxypropyl)pyrimidine-2,4(1*H*,3*H*)-dione (**16**) is mentioned in a patent [42], but it has not yet been characterised. We observed both monoalkylation (11%) and dialkylation (5%) in oil bath heating (Table 7, entry 1). The yields are not satisfactory, and the reaction under MW irradiation provided similar results with a more balanced product ratio. By increasing the reaction time to 7 h, the two compounds were produced in an equal amount of 13%.

**Scheme 15:**
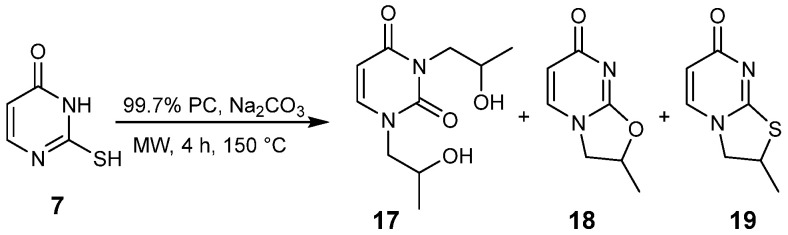
*N*-Alkylation of 2-thiouracil (**7**) with 99.7% PC without CaCl_2_.

Next, 99% PC was replaced by 99.7% PC and reactions were performed without CaCl_2_. Under these modified conditions, three products (**17**, **18** and **19**; Scheme 15) were detected with 1,3-bis(2-hydroxypropyl)pyrimidine-2,4(1*H*,3*H*)dione (**17**) in a significantly decreased yield of 7%. As already discussed, (Scheme 13) both 2-thiouracil and uracil are present in the alkaline solution. The high-quality PC increases the cyclisation rate (Table 7, entry 2) of the mono-*N*-alkylated product (**16**) to serve the following two new products: one with an oxazole ring (**18**, 34%) [43,44] and the other with a thiazole ring (**19**, 17%). By increasing the reaction time (Table 7, entry 3), the amount of both products **18** and **19** decreases somewhat (20% and 14%), while more dialkylated product can be obtained (**17**, 30%).

**Scheme 16:**
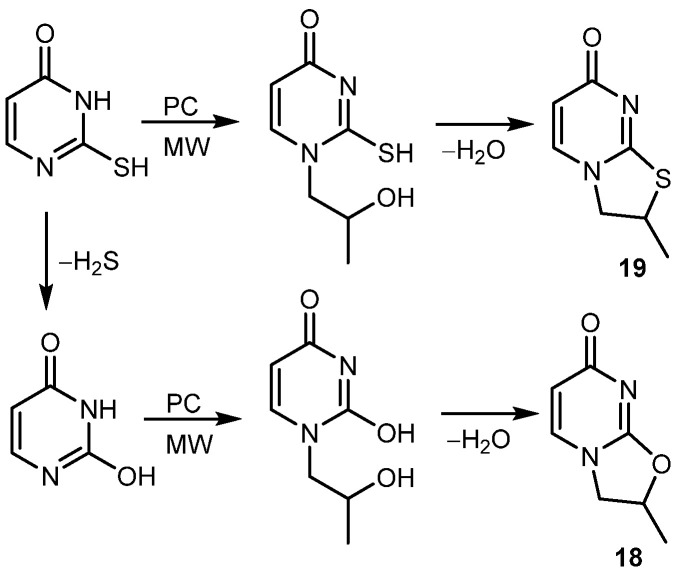
Cyclisation products of uracil and 2-thiouracil.

We have demonstrated that the sensible equilibrium, observed in previous studies performing alkylation of 2-thiouracil, exists under our conditions as well, serving different products. We assume that the cyclised compounds (**18**, **19**) can be obtained after monoalkylation has taken place on the 1*N* atom, followed by elimination of a water molecule from the alkylated side chain and the hydrogen of the hydroxy or thiol group attached to the C-2 atom (Scheme 16). Dialkylated product **17** and thiazole-condensed product **19** have not yet been published.

### 2.8. Theoretical Calculations

For the elucidation of the results obtained, theoretical calculations were carried out [44,45,46,47] and pKa values were determined for compounds **1**–**7**. In the case of **1**–**3**, there is only a single protic hydrogen in the molecules, attached to the nitrogen atom with calculated pKa values of 8.63, 9.70 and 11.89, respectively. These data indicate that under alkaline conditions, protons can be removed and the formed anions as nucleophiles are capable of opening PC, providing the *N*-alkylated product.

Considering compound **4**, there is only a single C–H in the molecule with a protic nature, but the two nitrogen atoms can also carry protic hydrogens. As a consequence, two products are expected. The pKa values calculated are 7.91 and 8.40 for 1*N* and 3*N*, respectively, indicating that the 1*N* position is more acidic. This corroborates our experimental results, since the 1*N*-alkylated product was formed with a larger amount. Importantly, no doubly alkylated product was observed.

Regarding compound **5**, both nitrogen atoms bear a hydrogen of protic nature; thus, double alkylation might be expected. The pKa values calculated are 9.44 and 9.34 for 1*N* and 3*N*, respectively. According to these data, the first alkylation may happen in position 3. The pKa value calculated for the 3*N*-alkylated derivative of **5** is 8.81. Accordingly, the alkylation of **5** in position 3 enhanced the acidity of position 1, and this explains why the doubly alkylated product was isolated as the sole derivative.

Concerning compound **6**, there is only a single hydrogen of protic character in the molecule. However, the anion formed after deprotonation has two mesomeric forms, with the negative charge located at either position 1 or position 2. Theoretical calculations suggest that the latter mesomeric form is slightly more stable (~1 kcal/mol); thus, charge transition is possible.

Finally, for compound **7,** results may be expected to be like those of 5. The pKa values calculated are 6.54 and 7.15 for 1*N* and 3*N*, respectively. Both protons have a significant acidic nature, and the observed double alkylation is in harmony with theoretical calculations.

The results of the reactions are summarised in Table 8.

The cyclised compounds (**18**, **19**) can be obtained only after monoalkylation has taken place on the 1*N* atom, followed by elimination of a water molecule.

## 3. Materials and Methods

**Experimental**

Phthalimide (99%) and propylene carbonate (99%) were purchased from Alfa Aesar. 1(2*H*)-Phthalazinone (99%), 4(3*H*)-pyrimidone (98%), 2,4-dihydroxy-6-methylpyrimidine (97%) and propylene carbonate (99.7%) were purchased from Sigma-Aldrich. Isatin (98%) was purchased from Reanal, 1*H*-benzotriazole (99%) was purchased from Merck, 2-thiouracil (98%) was purchased from Fluka, sodium carbonate (99.5%) was purchased from Acidum and calcium chloride (98.1%) was purchased from Molar.

Thin-layer chromatography (TLC) was performed on aluminium sheets precoated with Merck 5735 Kieselgel 60F254 (Merck, Darmastadt). Column chromatography was carried out with Merck 5735 Kieselgel 60F (0.040–0.063 nm mesh). All other chemicals and solvents were purchased from different commercial sources and used as received without further purification.

Freeze-drying was performed one night in a LYPH-Lock 1L lyophiliser LabConco (Kansas City, MI, USA) with a high vacuum pump at 10 mmHg and −50 °C. Melting points were measured on a Büchi M-550 apparatus (Büchi Labortechnik AG, Flawil, Switzerland) and are not corrected.

**Procedures**

**Method A: Reaction under oil bath (with 99% PC and drying agent)**

The substrate (4 mmol of **1**, **2**, **4**, **5**, **6** or **7**, except for **3**: 3 mmol), the solid Na_2_CO_3_ (4 mmol in the case of **1**, **2**, **4**, **5**, **6** or **7**, except for **3**: 3 mmol), the drying agent CaCl_2_ (4 mmol in the case of **1**, **2**, **4**, **5**, **6** or **7**, except for **3**: 3 mmol) and 99% propylene carbonate (36 mmol, 3 mL, d = 1.204 g/mL in the case of **1**, **2**, **3**, **4**, **6** or **7**, except for **5**: 48 mmol, 4 mL, d = 1.204 g/mL) were measured into a round-bottom flask with a Liebig-condenser and gas-outlet adapter and the suspension was treated at reflux temperature at a max. oil bath temperature of 170 °C. After the different reaction time (Tables S2–S8), the suspension was cooled down and the unreacted solid filtered off. After washing with water, the mother liquid was neutralised with 10% HCl solution and the aqueous layer was extracted with CHCl_3_ (3 × 25 mL, in the case of **2**, **5**) and EtOAc (3 × 25 mL, in the case of **1**, **3**, **4**, **6**, **7**), respectively. Usually, the organic phase contained the product (**10**, **11**, **12**, **16** and **17**), but in some cases, the extraction was satisfactory only to separate the unreacted propylene carbonate and propylene glycol from the raw product, which remained in the neutralised aqueous phase (product **8**, **9**, **13**, **14**, **15**, **18** and **19**). The collected organic phase was washed with 10% CuSO_4_ solution (2 × 15 mL) and evaporated after drying over Na_2_SO_4_ and filtration. In each case, the crude product was lyophilised overnight at 10 mmHg and −50 °C and weighted before the product was purified by column chromatography (silica gel, 0.040–0.063 mesh size, except product **10,** obtained after treatment with hexane). The unsuccessful reactions are not described in detail, but some are mentioned in Tables S2 and S3. All pure products—**8**, **9**, **10**, **11**, **12**, **13**, **14**, **15**, **16**, **17**, **18** and **19**—were characterised by ^1^H-, ^13^C-NMR spectroscopy and HPLC-MS.

**Method B: Reactions under MW conditions (with and without drying agent)**

MW-assisted experiments were carried out in a monomode CEM-Discover MW reactor using the standard configuration as delivered, including proprietary software. The experiments were executed in 80 mL MW process vials, a dynamic method with control of the temperature by infrared detection. Conditions: 5 min. ramp time, 150 °C temperature, different hold time, max. 200 Psi pressure and 300 W power. The amount of reagents was identical to that used in Method A; however, in spite of that, the use of drying agent was not necessary when 99.7% PC was the reagent and solvent too. After the corresponding reaction time (Tables S2–S8), the vial was cooled to 50 °C by air jet cooling, followed by the usual work-up, described in Method A.

Structure characterisation data:

72 mg (11%) yellowish oil **1-(2-hydroxypropyl)pyrimidine-2,4(1*H*,3*H*)-dione (16),** C_7_H_10_N_2_O_3_: 170.17, CAS Reg. No: 1479918-99-4, Rf = 0.40 (CHCl_3_/MeOH 5/1), rt = 0.23′ (94%), *m*/*z* = 171.

^1^H NMR (400 MHz, DMSO-*d_6_*): δ = 1.04 (d, *J* = 6.2 Hz, 3H, CH_3_), 3.38 (dd, *J* = 13.6, 8.4 Hz, 1H, CH_2_), 3.71 (dd, *J* = 13.6, 3.6 Hz, 1H, CH_2_), 3.82 (m, 1H, C*H*OH), 5.49 (d, *J* = 7.8 Hz, 1H, O=CCH), 7.52 (d, *J* = 7.8 Hz, 1H, NCH).

^13^C NMR (100 MHz, DMSO-*d_6_*): δ = 20.7, 54.5, 64.0, 100.0, 146.9, 151.3, 164.1.

42 mg (5%) white oily solid **1,3-bis(2-hydroxypropyl)pyrimidine-2,4(1*H*,3*H*)-dione (17),** C_10_H_16_N_2_O_4_: 228.25, Rf = 0.55 (CHCl_3_/MeOH 5/1), rt = 0.22′ (100%), *m*/*z* = 229.

^1^H NMR (400 MHz, DMSO-*d_6_*): δ = 1.00 (d, *J* = 6.0 Hz, 3H, N^3^CH_2_CHC*H_3_*), 1.05 (d, *J* = 6.2 Hz, 3H, N^1^CH_2_CHC*H_3_*), 3.44 (m, 1H, N^1^CH_2_), 3.66 (m, 1H, N^3^CH_2_), 3.77 (m, 1H, N^1^CH_2_), 3.84 (m, 1H, N^3^CH_2_), 3.83 (m, 1H, N^1^CH_2_C*H*), 3.89 (m, 1H, N^3^CH_2_C*H*), 4.67 (d, *J* = 5.2 Hz, 1H, N^3^CH_2_CHO*H*), 4.93 (d, *J* = 4.8 Hz, 1H, N^1^CH_2_CHO*H*) 5.63 (d, *J* = 7.8 Hz, 1H, O=CCH), 7.54 (d, *J* = 7.8 Hz, 1H, NCH).

^13^C NMR (100 MHz, DMSO-*d_6_*): δ = 20.7, 21.1, 47.1, 55.7, 63.3, 64.0, 99.4, 145.3, 151.5, 162.9.

## 4. Conclusions

We have studied the *N*-alkylation power of PC functioning as both an alkylating agent and solvent, under alkaline conditions (solid Na_2_CO_3_), either without the use (anhydrous PC (99.7%)) or in the presence of a drying agent (CaCl_2_, 99% PC) in one-pot reactions. In each case, *N*-alkylation took place, and we were able to prepare new monoalkylated compounds **12** and **14**. In the case of 6-methyluracil (**5**) and 2-thiouracil (**7**), dialkylation occurs under our reaction conditions, suggesting that the electron-donating methyl and thiol groups activate the heterocyclic system towards the electrophilic carbon atom of PC. In the case of 2-thiouracil (**7**), both dialkylation and monoalkylation were observed, delivering new compounds **9** and **19**, in addition to the monoalkylated product **8** and the oxazole-condensed ring system **18**. It is suggested that the most productive *N*-alkylation is achieved with the use of anhydrous PC under MW conditions. We were also able to transform compound **7** into two cyclised compounds (**18**, **19**) by dehydration. *N*-Alkylation was also successful in the case of benzotriazole (**6**), synthesizing two monoalkylated products. This is despite the lack of the electron-withdrawing carbonyl group. Finally, the observed experimental data were supported by theoretical calculations.

## Figures and Tables

**Table 1:** *N*-Hydroxyalkylation of phthalimide (**1**).

Entry	Heating	PC (mmol/%)	Base	T (°C)	Time (h)	Drying Agent	Yield (%) 8
1	MW	6/99%	1 mmol 1M Na_2_CO_3_	130	2	none	-
2	MW	9/99%	1 mmol 1M Na_2_CO_3_	130	2	100 mg MS (3 Å)	49
3	MW	9/99%	1 mmol Na_2_CO_3_^a^	150	1	1 mmol CaCl_2_	63
4	MW	9/99.7%	1 mmol Na_2_CO_3_^a^	150	1	none	70
5	oil bath	9/99%	1 mmol Na_2_CO_3_^a^	170	4	1 mmol CaCl_2_	66

^a^ Aqueous or powder.

**Table 2:** *N*-Hydroxyalkylation of isatine (**2**).

Entry	Heating	PC (mmol/%)	Na_2_CO_3_	T (°C)	Time (h)	CaCl_2_	Yield (%) 9
1	MW	9/99%	1 mmol	160	1	1 mmol	-
2	MW	9/99.7%	1 mmol	150	1	none	77

**Table 3:** *N*-Hydroxyalkylation of phthalazin-1(2*H*)-one (**3**).

Entry	Heating	PC (mmol/%)	Na_2_CO_3_	T (°C)	Time (h)	CaCl_2_	Yield (%) 10
1	oil bath	12/99%	1 mmol	170	4	1 mmol	28
2	MW	12/99%	1 mmol	150	4	1 mmol	50
3	MW	12/99.7%	1 mmol	150	2	none	55

**Table 4:** *N*-Hydroxyalkylation of pyrimidin-4(3*H*)-one (**4**).

Entry	Heating	PC (mmol/%)	Na_2_CO_3_	T (°C)	Time (h)	CaCl_2_	Yield (%) 11,12
1	oil bath	9/99%	1 mmol	170	3	1 mmol	28, 54
2	MW	9/99.7%	1 mmol	150	1	none	42, 57

**Table 5:** Results with 6-methylpyrimidine-2,4(1*H*,3*H*)-dione (**5**).

Entry	Heating	PC (mmol/%)	Na_2_CO_3_	T (°C)	Time (h)	CaCl_2_	Yield (%) 13
1	MW	9/99%	1 mmol	150	6	1 mmol	12
2	MW	9/99.7%	1 mmol	150	2	none	22
3	MW	9/99.7%	1 mmol	150	6	none	49
4	oil bath	12/99%	1 mmol	170	9	1 mmol	6

**Table 6:** *N*-Alkylation of 1*H*-benzotriazole (**6**).

Entry	Heating	PC (mmol/%)	Na_2_CO_3_	T (°C)	Time (h)	CaCl_2_	Yield (%) 14, 15
1	oil bath	9/99%	1 mmol	170	3	1 mmol	22, 47
2	MW	9/99%	1 mmol	150	3	1 mmol	25, 53
3	MW	9/99.7%	1 mmol	150	4	none	35, 55

**Table 7:** *N*-Alkylation of 2-thiouracil (**7**).

Entry	Heating	PC (mmol/%)	Na_2_CO_3_	T (°C)	Time (h)	CaCl_2_	Yield (%) 16, 17, 18, 19
1	oil bath	9/99%	1 mmol	170	3	1 mmol	11, 5, n.i., n.i.
2	MW	9/99.7%	1 mmol	150	2	none	n.i., 7, 34, 17
3	MW	9/99.7%	1 mmol	150	4	none	n.i., 30, 20, 14

**Table 8:** Product yields under optimised reaction conditions.

Reactant	Product(s) Yield (%)
1	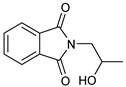 **8:** 70%
2	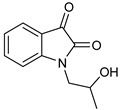 **9:** 77%
3 [a]	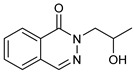 **10:** 55%
4	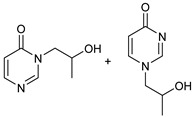 **11:** 42% **12:** 57%
5 [b]	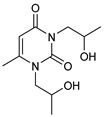 **13:** 49%
6 [c]	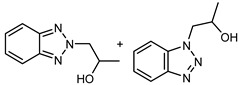 **14:** 35% **15:** 55%
7 [c]	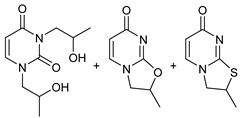 **17:** 30% **18:** 20% **19:** 14%

[a]: reaction time 2 h, [b]: reaction time 6 h, [c]: reaction time 4 h.

## Data Availability

Data are contained within the article or Supplementary Materials.

## Supplementary Materials

The following supporting information can be downloaded at: https://www.mdpi.com/article/10.3390/ijms25105523/s1.
