# Supplementary material for: Sustainable and Safe N-alkylation of N-heterocycles by Propylene Carbonate under Neat Reaction Conditions
Source: Int J Mol Sci. 2024 May 18;25(10):5523. doi: 10.3390/ijms25105523 (PMC11122188; doi:10.3390/ijms25105523)
Supplement: Supplementary file 1 [file ijms-25-05523-s001.zip › ijms-2997680-supplementary.pdf]

Sustainable and Safe *N*-alkylation of *N*-heterocycles by Propylene Carbonate under Neat Reaction Conditions

Andrea Czompa [1], Dóra Bogdán [1,2], Balázs Balogh [1], Eszter Erdei [1,2], Patrik Selymes [1], Attila Csomos [3,4] and István M. Mándity [1,2,*]

[1] Institute of Organic Chemistry, Semmelweis University, Hőgyes Endre utca 7, H-1092 Budapest, Hungary; czompa.andrea@semmelweis.hu (A.C.); bogdan.dora@semmelweis.hu (D.B.); balogh.balazs@semmelweis.hu (B.B.); erdei.eszter@stud.semmelweis.hu (E.E.); patrik.selymes@gmail.com (P.S.)

[2] HUN-REN Artificial Transporters Research Group, Institute of Materials and Environmental Chemistry, Research Centre for Natural Sciences Magyar Tudósok körútja 2, H-1117 Budapest, Hungary

[3] Femtonics Ltd., Tüzoltó utca 59, H-1094 Budapest, Hungary; attila.csomos@femtonics.eu

[4] Hevesy György PhD School of Chemistry, Eötvös Loránd University, Pázmány Péter sétány 1/A, H-1117 Budapest, Hungary

* Correspondence: mandity.istvan@ttk.hu; Tel.: +36-205-230-081

Contents

Experimental section 3

HPLC-MS data, ^{1}H- and ^{13}C-NMR spectrum

2-(2-hydroxypropyl)-1*H*-isoindole-1,3(2*H*)-dione (8) 10

1-(2-hydroxypropyl)-1*H*-indole-2,3-dione (9) 11

2-(2-hydroxypropyl)phthalazin-1(2*H*)-one (10) 13

3-(2-hydroxypropyl)pyrimidin-4(3*H*)-one (11) 14

1-(2-hydroxypropyl)pyrimidin-4(1*H*)-one (12) 16

1,3-bis(2-hydroxypropyl)-6-methylpyrimidine-2,4(1*H*,3*H*)-dione (13) 17

2-(2-hydroxypropyl)benzotriazole (14) 19

1-(2-hydroxypropyl) benzotriazole (15) 20

1-(2-hydroxypropyl)pyrimidine-2,4(1*H*,3*H*)-dione (16) 22

1,3-bis(2-hydroxypropyl)pyrimidine-2,4(1*H*,3*H*)-dione (17) 23

2-methyl-2,3-dihydro-[1,3]oxazolo[3,2-a]pyrimidin-7-one (18) 25

2-methyl-2,3-dihydro-[1,3]thiazolo[3,2-a] pyrimidin-7-one (19) 26

Theoretical calculations 28

Discussion 30

Experimental section

Phthalimide (99%) and propylene carbonate (99%) were purchased from Alfa Aesar. 1(2*H*)-Phthalazinone (99%), 4(3*H*)-pyrimidone (98%), 2,4-dihydroxy-6-methylpyrimidine (97%) and propylene carbonate (99.7%) were purchased from Sigma-Aldrich. Isatin (98%) was purchased from Reanal, 1*H*-benzotriazole (99%) from Merck, 2-thiouracil (98%) from Fluka, while sodium carbonate (99.5%) was purchased from Acidum and calcium chloride (98.1%) from Molar.
Thin-layer chromatography (TLC) was performed on aluminium sheets precoated with Merck 5735 Kieselgel 60F254. Column chromatography was carried out either with Merck 5735 Kieselgel 60F (0.040–0.063 nm mesh). All other chemicals and solvents were purchased from different commercial sources and used as received without further purification.
Freeze-drying was performed one night in a LYPH-Lock 1L lyophilizer LabConco (Kansas City, Missouri) with high vacuum pump, at 10 mmHg and –50 °C. Melting points were measured on a Büchi M-550 apparatus (Büchi Labortechnik AG, Switzerland) and are not corrected.

NMR measurements

^{1}H (400 MHz) and ^{13}C (100 MHz) NMR spectra were recorded at room temperature on Varian Mercury Plus spectrometer. Amounts of 10–15 mg of compounds were dissolved in 0.6 ml DMSO-d_6 or a mixture of DMSO-d_6 + D_2O and transferred to 5 mm NMR sample tubes. Chemical shifts are given on the δ-scale and referenced to the solvent (DMSO-d_6: δ_C=39.50 ppm and δ_H=2.50 ppm). For ^{1}H NMR measurement 24 K data points, 2.0 s acquisition time and 6400 Hz sweep width were used. ^{13}C spectra were recorded with 62 K data points and 24000 Hz sweep width. For 2D measurements, in case of the gHSQC spectrum the sweep width in F2 was 3000 Hz; data points (t2 × t1) were acquired with 1 K × 128, in case of the gHMBC spectrum the sweep width in F2 was 3000 Hz; data points (t2 × t1) were acquired with 1 K × 256, respectively.

HPLC-MS method used for the determination of product purity

The HPLC-MS system consisted of Shimadzu LC-40AD XR parallel pumps, with Shimadzu DGU-405 degasser unit, Shimadzu SIL-40C XR Autosampler, CTO-40S thermostat at 40 °C, and LCMS-2020 mass spectrometer, equipped with DUIS ionizer (Drying gas 15L/min, Nebulizing gas 1.5 L/min; the DL temp is 250 °C, ESI 10 000 V). The HPLC column was an Ascentis C-18 2 μm, 2.1 × 5 mm. Eluent A was 0.1% TFA in water (from Merck); eluent B was 0.1% TFA in acetonitrile (from Merck). The gradient was set by a linear program, where eluent B was increased from 0% to 100% within 4.5 min (Table S1). The flow rate was 0.75 ml/min. The obtained retention times measured at 254 nm are specified at the corresponding compound.

Table S1. Gradient elution in HPLC-MS method

Time (min.)	A(%)	B(%)
**0.0**	95.0	5.0
**0.5**	0.0	100.0

A: water with 0.1% TFA; B: acetonitrile with 0.1% TFA.

Procedures

Method A: Reaction under oil-bath (with 99% PC and drying agent)

The substrate (4 mmol of **1**, **2**, **4**, **5**, **6** or **7**, except for **3**: 3 mmol), the solid Na_2CO_3 (4 mmol in the case of **1**, **2**, **4**, **5**, **6** or **7**, except for **3**: 3 mmol), the drying agent $CaCl_2$ (4 mmol in the case of **1**, **2**, **4**, **5**, **6** or **7**, except for **3**: 3 mmol), 99 % propylene carbonate (36 mmol, 3 mL, d = 1.204 g/mL in the case of **1**, **2**, **3**, **4**, **6** or **7**, except for **5**: 48 mmol, 4 mL, d = 1.204 g/mL) was measured into a round-bottom flask with Liebig-condenser

and gas-outlet adapter and the suspension was treated at reflux temperature at a max. oil-bath temperature of 170 °C. After the different reaction time (Table S2, S3, S4, S5, S6, S7, S8), the suspension was cooled down and the unreacted solid filtered off. After washing with water, the mother liquid was neutralized with 10% HCl solution and the aqueous layer was extracted with $CHCl_3$ (3 × 25 mL, in the case of **2**, **5**), respectively EtOAc (3 × 25 mL, in the case of **1**, **3**, **4**, **6**, **7**). Usually, the organic phase contained the product (**10**, **11**, **12**, **16** and **17**), but in some cases, the extraction was satisfactory only to separate the unreacted propylene carbonate and propylene glycol from the raw product, which remained in the neutralized aqueous phase (product **8**, **9**, **13**, **14**, **15**, **18** and **19**). The collected organic phase was washed with 10% $CuSO_4$ solution (2 × 15 mL) and evaporated, after drying over Na_2SO_4 and filtration. In each case, the crude product was lyophilized overnight at 10 mmHg and –50 °C and weighted, before the product was purified by column chromatography (silica gel, 0.040–0.063 mesh size, except product **10** obtained after treatment with hexane). The unsuccessful reactions are not described in details, but some are mentioned in Table S2 and S3. All pure products: **8**, **9**, **10**, **11**, **12**, **13**, **14**, **15**, **16**, **17**, **18** and **19** were characterized by ^{1}H-, ^{13}C-NMR spectroscopy and HPLC-MS.

Method B: Reactions under MW conditions (with and without drying agent)
MW assisted experiments were carried out in a monomode CEM-Discover MW reactor, using the standard configuration as delivered, including proprietary software. The experiments were executed in 80 mL MW process vials, dynamic method with control of the temperature by infrared detection. Conditions: 5 min. ramp rime, 150 °C temperature, different hold time, max. 200 Psi pressure and 300 W power. The amount of reagents was identical with that used in Method A, in spite of that the use of drying agent was not necessary when 99.7 % PC was the reagent and solvent too. After the corresponding reaction time (Table S2, S3, S4, S5, S6, S7, S8), the vial was cooled to 50 °C by air jet cooling, followed by usual work-up, described in Method A.

2-(2-hydroxypropyl)-1*H*-isoindole-1,3(2*H*)-dione (8)

Table S2. *N*-Alkylation of phthalimide (**1**)

Entry	Heating	PC (mmol/%)	Na_2CO_3	T (°C)	Time (h)	Drying agent	Yield (%) **8**
1	oil bath	36/99%	4 mmol	170	4	4 mmol $CaCl_2$	66
2		24/99%	4 mmol 1M Na_2CO_3	130	2	none	–
3	MW	36/99%				400 mg MS (3 Å)	49
4		36/99%	4 mmol	150	1	4 mmol $CaCl_2$	63
5		36/99.7%				none	70

Table S2, entry 1: The 730 mg crude product obtained after lyophilization of the organic phase (EtOAc) was treated with 2 × 5 ml hexane, filtered and dried until constant weight:
539 mg (66%) off-white solid **2-(2-hydroxypropyl)-1*H*-isoindole-1,3(2*H*)-dione (8),** $C_{11}H_{11}NO_3$: 205.21, CAS Reg. No: 3700-55-8, Rf = 0.58 ($CHCl_3$/EtOAc 1/1), Mp: 86.9-89.1°C, rt = 4.78' (92%), m/z: 206.
^{1}H NMR (400 MHz, DMSO-d_6): δ = 1.07 (d, J = 6.3Hz, 3H, CH_3), 3.41 (dd, J = 13.6, 4.9Hz, 1H, CH_2), 3.53 (dd, J = 13.6, 8.1Hz, 1H, CH_2), 3.92 (m, 1H, C*H*OH), 7.90-7.80 (m, 4H, ArCH).
^{13}C NMR (100 MHz, DMSO-d_6): δ = 20.9, 45.3, 63.5, 122.9, 131.8, 134.3, 168.1.

Table S2, entry 3: The 580 mg crude product obtained after lyophilization of the organic phase (EtOAc) was treated with 2 × 5 ml hexane, filtered and dried until constant weight: 403 mg (49%).
Table S2, entry 4: The 810 mg crude product obtained after lyophilisation of the organic phase (EtOAc) was treated with 2 × 5 ml hexane, filtered and dried until constant weight: 516 mg (63%).
Table S2, entry 5: The 880 mg crude product obtained after lyophilisation of the organic phase (EtOAc) was treated with 2 × 5 ml hexane, filtered and dried until constant weight: 575 mg (70%).

1-(2-hydroxypropyl)-1*H*-indole-2,3-dione (9)

Table S3. *N*-Alkylation of isatin (**2**)

Entry	Heating	PC (mmol/%)	Na_2CO_3	T (°C)	Time (h)	$CaCl_2$	Yield (%) **9**
1	MW	36/99%	4 mmol	160	1	4 mmol	–
2		36/99.7%		150	1	none	77

Table S3, entry 2: The 993 mg crude product obtained after lyophilization of the organic phase ($CHCl_3$) was purified by column chromatography on 10 g silica gel (0.040–0.063 mesh size), with gradient elution: $CHCl_3$, followed by $CHCl_3$/EtOAc 2/1 and 1/1. The fractions containing the pure product were collected, evaporated and dried until constant weight:
634 mg (77%) red solid, $C_{11}H_{11}NO_3$: 205.21, CAS Reg. No: 503855-32-1, Rf = 0.33 ($CHCl_3$/EtOAc 1/1), Mp: 66.5-68.3 °C, rt = 4.45' (95%), m/z = 206.
^{1}H NMR (400 MHz, DMSO-*d*$_6$): δ = 1.12 (d, *J* = 6.3Hz, 3H, CH_3), 3.64-3.51 (m, 2H, CH_2), 3.96 (m, 1H, C*H*OH), 4.92 (d, *J* = 4.3Hz, 1H, OH), 7.11 (t, *J* = 7.8Hz, 1H, H-5), 7.21 (d, *J* = 7.8Hz, 1H, H-7), 7.53 (d, *J* = 7.8Hz, 1H, H-4), 7.64 (t, *J* = 7.8Hz, 1H, H-6).
^{13}C NMR (100 MHz, DMSO-*d*$_6$): δ = 20.7, 47.4, 63.5, 111.4, 117.5, 122.9, 124.1, 137.9, 151.5, 158.4, 183.7.

2-(2-hydroxypropyl)phthalazin-1(2*H*)-one (10)

Table S4. *N*-Alkylation of phthalazin-1(2*H*)-one (**3**)

Entry	Heating	PC (mmol/%)	Na_2CO_3	T (°C)	Time (h)	$CaCl_2$	Yield (%) **10**
1	oil bath	36/99%		170	4	3 mmol	28
2	MW	36/99%	3 mmol	150	4	3 mmol	50
3		36/99.7%		150	2	none	55

Table S4, entry 1: The 620 mg crude product obtained after lyophilization of the organic phase (EtOAc) was purified by column chromatography on 10 g silica gel (0.040–0.063 mesh size), with gradient elution: $CHCl_3$, followed by $CHCl_3$/EtOAc 1/1. The fractions containing the pure product were collected, evaporated and dried until constant weight:
170 mg (28%) brownish oil **2-(2-hydroxypropyl)phthalazin-1(2*H*)-one (10),** $C_{11}H_{12}N_2O_2$: 204.23, CAS Reg. No: 1250877-71-4, Rf = 0.38 ($CHCl_3$/EtOAc 1/1), rt = 2.85' (95%), m/z = 205.
^{1}H NMR (400 MHz, DMSO-*d*$_6$): δ = 1.09 (d, *J* = 5.9Hz, 3H, CH_3), 3.97 (m, 1H, CH_2), 4.20-4.05 (m, 2H, CH_2 and C*H*OH), 4.81 (d, *J* = 4.7Hz, 1H, OH), 7.85 (m, 1H, H-7), 7.97-7.89 (m, 2H, H-5 and H-6), 8.25 (d, *J* = 7.8Hz, 1H, H-8), 8.41 (s, 1H, NCH).
^{13}C NMR (100 MHz, DMSO-*d*$_6$): δ = 21.0, 57.5, 64.1, 125.8, 126.7, 127.2, 129.3, 131.9, 133.4, 137.6, 158.7.

Table S4, entry 2: The 603 mg crude product obtained after lyophilization of the organic phase (EtOAc) was purified by column chromatography on 10 g silica gel (0.040–0.063 mesh size), with gradient elution: $CHCl_3$, followed by $CHCl_3$/EtOAc 1/1. The fractions containing the pure product were collected, evaporated and dried until constant weight: 309 mg (50%).
Table S4, entry 3: The 844 mg crude product obtained after lyophilization of the organic phase (EtOAc) was purified by column chromatography on 10 g silica gel (0.040–0.063 mesh size), with gradient elution: $CHCl_3$, followed by $CHCl_3$/EtOAc 1/1. The fractions containing the pure product were collected, evaporated and dried until constant weight: 336 mg (55%).

3-(2-hydroxypropyl)pyrimidin-4(3*H*)-one (11) and 1-(2-hydroxypropyl)pyrimidin-4(1*H*)-one (12)

Table S5. *N*-Alkylation of pyrimidin-4(3*H*)-one (**4**)

Entry	Heating	PC (mmol/%)	Na_2CO_3	T (°C)	Time (h)	$CaCl_2$	Yield (%) **11**, **12**
1	oil bath	36/99%	4 mmol	170	3	4 mmol	28, 54
2	MW	36/99.7%		150	1	none	42, 57

Table S5, entry 1: The 1.20 g crude product obtained after lyophilization of the aqueous phase was purified by column chromatography on 10 g silica gel (0.040–0.063 mesh size), with gradient elution: Toluene/MeOH 4/1, 2/1 and 1/1. The fractions containing the pure products were collected, evaporated and dried until constant weight:

174 mg (28%) yellow oil **3-(2-hydroxypropyl)pyrimidin-4(3*H*)-one (11),** $C_7H_{10}N_2O_2$: 154.17, CAS Reg. No: 1405334-56-6, Rf = 0.63 (Toluene/MeOH 1/1), rt = 0.21' (100%), m/z = 155.

^{1}H NMR (400 MHz, DMSO-*d*$_6$): δ = 1.08 (d, *J* = 6.3Hz, 3H, CH_3), 3.56 (dd, *J* = 13.2, 8.5Hz, 1H, CH_2), 3.85 (m, 1H, C<u>*H*</u>OH), 4.00 (dd, *J* = 13.2, 3.1Hz, 1H, CH_2), 5.00 (brs, 1H, OH), 6.38 (d, *J* = 6.6 Hz, 1H, HCC=O), 7.89 (d, *J*=6.6Hz, 1H, NCH), 8.29 (s, 1H, NCHN).

^{13}C NMR (100 MHz, DMSO-*d*$_6$): δ = 21.0, 52.9, 63.3, 114.8, 153.3, 153.6, 160.4.

335 mg (54%) yellow amorph solid **1-(2-hydroxypropyl)pyrimidin-4(1*H*)-one (12),** $C_7H_{10}N_2O_2$: 154.17, Rf = 0.38 (Toluene/MeOH 1/1), Mp > 270 °C (decomp.), rt = 0.56' (97%), m/z = 155 and 331 [2M + Na].

^{1}H NMR (400 MHz, DMSO-*d*$_6$): δ = 1.05 (d, *J* = 6.2Hz, 3H, CH_3), 3.64 (dd, *J* = 13.8, 8.0Hz, 1H, CH_2), 3.82 (m, 1H, C<u>*H*</u>OH), 3.90 (m, 1H, CH_2), 6.08 (d, *J* = 7.6 Hz, 1H, O=CCH), 7.73 (dd, *J* = 7.6, 2.5Hz, 1H, NCH), 8.23 (d, *J* = 2.5Hz, 1H, NCHN).

^{13}C NMR (100 MHz, DMSO-*d*$_6$): δ = 20.7, 60.0, 65.9, 111.4, 144.9, 154.3, 171.3.

Table S5, entry 2: The 800 g crude product obtained after lyophilization of the aqueous phase was purified by column chromatography on 10 g silica gel (0.040–0.063 mesh size), with gradient elution: Toluene, followed by Toluene/MeOH 2/1 and 1/1. The fractions containing the pure products were collected, evaporated and dried until constant weight: 260 mg (42%) product **11** and 350 mg (57%) product **12**.

1,3-bis(2-hydroxypropyl)-6-methylpyrimidine-2,4(1*H*,3*H*)-dione (13)

Table S6. Results with 6-methylpyrimidine-2,4(1*H*,3*H*)-dione (**5**)

Entry	Heating	PC (mmol/%)	Na_2CO_3	T (°C)	Time (h)	$CaCl_2$	Yield (%) **13**
1		36/99%			6	4 mmol	12
2	MW	36/99.7%	4 mmol	150	2	none	22
3		36/99.7%			6	none	49

Table S6, entry 1: The 1.13 g crude product obtained after lyophilization of the aqueous phase was purified by column chromatography on 10 g silica gel (0.040–0.063 mesh size), with gradient elution: Toluene, followed by Toluene/MeOH 5/1, 2/1 and 1/1. The fractions containing the pure product were collected, evaporated,

and dried until constant weight:

124 mg (12%) white solid **1,3-bis(2-hydroxypropyl)-6-methylpyrimidine-2,4(1*H*,3*H*)-dione (13),** $C_{11}H_{18}N_2O_4$: 242.27, CAS Reg. No: 2305764-32-1, Rf = 0.63 ($CHCl_3$/MeOH 5/1), Mp: 139.2-141.6 °C, rt = 3.62' (100%), m/z = 243.

^{1}H NMR (400 MHz, DMSO-*d*$_6$): δ = 0.99 (d, *J* = 5.6Hz, 3H, $N^3CH_2CHC\underline{H_3}$), 1.08 (d, *J* = 6.3Hz, 3H, $N^1CH_2CHC\underline{H_3}$), 2.28 (s, 3H, C^6CH_3), 3.52 (m, 1H, N^1CH_2), 3.65 (m, 1H, N^3CH_2), 3.77 (m, 1H, N^1CH_2), 3.82

(m, 1H, N^3CH_2), 3.95-3.85 (m, 2H, $N^1CH_2C\underline{H}$ and $N^3CH_2C\underline{H}$), 4.64 (t, J = 5.4Hz, 1H, $N^3CH_2CHO\underline{H}$), 4.97 (t, J = 4.2Hz, 1H, $N^1CH_2CHO\underline{H}$), 5.57 (s, 1H, H-5).
^{13}C NMR (100 MHz, DMSO-d_6): δ =20.1, 21.0, 21.2, 47.2, 51.7, 63.4, 64.0, 100.1, 152.0, 153.7, 161.8.

Table S6, entry 2: The 863 mg crude product obtained after lyophilization of the aqueous phase was purified by column chromatography on 10 g silica gel (0.040–0.063 mesh size), with gradient elution: Toluene, followed by Toluene/MeOH 5/1, 2/1 and 1/1. The fractions containing the pure product were collected, evaporated and dried until constant weight: 215 mg (22%).
Table S6, entry 3: The 962 g crude product obtained after lyophilization of the aqueous phase was purified by column chromatography on 10 g silica gel (0.040–0.063 mesh size), with gradient elution: Toluene, followed by Toluene/MeOH 5/1, 2/1 and 1/1. The fractions containing the pure product were collected, evaporated and dried until constant weight: 476 mg (49%).

2-(2-hydroxypropyl)benzotriazole (14) and 1-(2-hydroxypropyl) benzotriazole (15)

Table S7. *N*-Alkylation of 1*H*-benzotriazole (**6**)

Entry	Heating	PC (mmol/%)	Na_2CO_3	T (°C)	Time (h)	$CaCl_2$	Yield (%) **14**, **15**
1	oil bath	36/99%		170	3	4 mmol	22, 47
2	MW	36/99%	4 mmol	150	3	4 mmol	25, 53
3		36/99.7%		150	4	none	35, 55

Table S7, entry 1: The 675 mg crude product obtained after lyophilization of the organic phase (EtOAc) was purified by column chromatography on 10 g silica gel (0.040–0.063 mesh size), with gradient elution: Toluene/EtOAc 2/1 and 1/1. The fractions containing the pure products were collected, evaporated and dried until constant weight:
154 mg (22%) brownish oil **2-(2-hydroxypropyl)benzotriazole (14),** $C_9H_{11}N_3O$: 177.21, CAS Reg. No: 90887-06-2, Rf = 0.60 (Toluene/EtOAc 1/1), rt = 2.99' (95%), m/z = 178.
^1H NMR (400 MHz, DMSO-d_6): δ = 1.15 (d, J = 6.3Hz, 3H, CH_3), 4.32 (m, 1H, $C\underline{H}OH$), 4.69-4.60 (m, 2H, CH_2), 5.06 (d, J = 4.8Hz, 1H, OH), 7.42 (m, 2H, H-4 and H-7), 7.91 (m, 2H, H-5 and H-6).
^{13}C NMR (100 MHz, DMSO-d_6): δ = 21.0, 63.1, 65.7, 117.8, 126.2, 143.7.
332 mg (47%) yellow oil **1-(2-hydroxypropyl) benzotriazole (15),** $C_9H_{11}N_3O$: 177.21, CAS Reg. No: 82131-97-3 [9, 10], Rf = 0.43 (Toluene/EtOAc 1/1), rt = 0.22' (97%), m/z = 178.
^1H NMR (400 MHz, DMSO-d_6): δ = 1.13 (d, J = 6.3Hz, 3H, CH_3), 4.14 (m, 1H, $C\underline{H}OH$), 4.57 (dd, J = 14.2, 7.2Hz, 1H, CH_2), 4.66 (dd, J = 14.2, 4.4Hz, 1H, CH_2), 5.03 (d, J = 5.0Hz, 1H, OH), 7.38 (t, J = 7.8Hz, 1H, H-5), 7.52 (t, J = 7.8Hz, 1H, H-6), 7.87 (d, J = 7.8Hz, 1H, H-7), 8.02 (d, J = 7.8Hz, 1H, H-4).
^{13}C NMR (100 MHz, DMSO-d_6): δ = 20.9, 54.8, 65.6, 111.4, 118.9, 123.7, 126.9, 133.7, 145.1.

Table S7, entry 2: The 724 mg crude product obtained after lyophilization of the organic phase (EtOAc) was purified by column chromatography on 10 g silica gel (0.040–0.063 mesh size), with gradient elution: Toluene/EtOAc 2/1 and 1/1. The fractions containing the pure products were collected, evaporated and dried until constant weight: 177 mg (25%) product **14** and 376 mg (53%) product **15**.
Table S7, entry 3: The 790 mg crude product obtained after lyophilization of the organic phase (EtOAc) was purified by column chromatography on 10 g silica gel (0.040–0.063 mesh size), with gradient elution: Toluene/EtOAc 2/1 and 1/1. The fractions containing the pure products were collected, evaporated and dried until constant weight: 247 mg (35%) product **14** and 390 mg (55%) product **15**.

1-(2-hydroxypropyl)pyrimidine-2,4(1*H*,3*H*)-dione (16) and 1,3-bis(2-hydroxypropyl) pyrimidine-2,4(1*H*,3*H*)-dione (17), 2-methyl-2,3-dihydro-[1,3] oxazolo[3,2-a]pyrimidin-7-one (18) and 2-methyl-2,3-dihydro-[1,3]thiazolo[3,2-a] pyrimidin-7-one (19)

Table S8. *N*-Alkylation of 2-thiouracil (**7**)

Entry	Heating	PC (mmol/%)	Na_2CO_3	T (°C)	Time (h)	$CaCl_2$	Yield (%) **16, 17, 18, 19**
1	oil bath	36/99%	4 mmol	170	2	4 mmol	11, 5, n.i., n.i.
2		36/99%		170	5	4 mmol	13, 13, n.i., n.i.
3	MW	36/99.7%		150	2	none	n.i., 7, 34, 17
4		36/99.7%		150	4	none	n.i., 30, 20, 14

Table S8, entry 1: The 320 mg crude product obtained after lyophilization of the aqueous phase was purified by column chromatography on 10 g silica gel (0.040–0.063 mesh size), with gradient elution: $CHCl_3$/MeOH 5/1, 2/1 and 1/1. The fractions containing the pure products were collected, evaporated and dried until constant weight:

72 mg (11%) yellowish oil **1-(2-hydroxypropyl)pyrimidine-2,4(1*H*,3*H*)-dione (16),** $C_7H_{10}N_2O_3$: 170.17, CAS Reg. No: 1479918-99-4, Rf = 0.40 ($CHCl_3$/MeOH 5/1), rt = 0.23’ (94%), m/z = 171.

^{1}H NMR (400 MHz, DMSO-d_6): δ = 1.04 (d, *J* = 6.2Hz, 3H, CH_3), 3.38 (dd, *J* = 13.6, 8.4Hz, 1H, CH_2), 3.71 (dd, *J* = 13.6, 3.6Hz, 1H, CH_2), 3.82 (m, 1H, C*H*OH), 5.49 (d, *J* = 7.8Hz, 1H, O=CCH), 7.52 (d, *J* = 7.8Hz, 1H, NCH).

^{13}C NMR (100 MHz, DMSO-d_6): δ = 20.7, 54.5, 64.0, 100.0, 146.9, 151.3, 164.1.

42 mg (5%) white oily solid **1,3-bis(2-hydroxypropyl)pyrimidine-2,4(1*H*,3*H*)-dione (17),** $C_{10}H_{16}N_2O_4$: 228.25, Rf = 0.55 ($CHCl_3$/MeOH 5/1), rt = 0.22’ (100%), m/z = 229.

^{1}H NMR (400 MHz, DMSO-d_6): δ = 1.00 (d, *J* = 6.0Hz, 3H, $N^3CH_2CHC\underline{H_3}$), 1.05 (d, *J* = 6.2Hz, 3H, $N^1CH_2CHC\underline{H_3}$), 3.44 (m, 1H, N^1CH_2), 3.66 (m, 1H, N^3CH_2), 3.77 (m, 1H, N^1CH_2), 3.84 (m, 1H, N^3CH_2), 3.83 (m, 1H, $N^1CH_2C\underline{H}$), 3.89 (m, 1H, $N^3CH_2C\underline{H}$), 4.67 (d, *J* = 5.2Hz, 1H, $N^3CH_2CHO\underline{H}$), 4.93 (d, *J* = 4.8Hz, 1H, $N^1CH_2CHO\underline{H}$) 5.63 (d, *J* = 7.8Hz, 1H, O=CCH), 7.54 (d, *J* = 7.8Hz, 1H, NCH).

^{13}C NMR (100 MHz, DMSO-d_6): δ = 20.7, 21.1, 47.1, 55.7, 63.3, 64.0, 99.4, 145.3, 151.5, 162.9.

Table S8, entry 2: The 728 mg crude product obtained after lyophilization of the aqueous phase was purified by column chromatography on 10 g silica gel (0.040–0.063 mesh size), with gradient elution: $CHCl_3$/MeOH 5/1, 2/1 and 1/1. The fractions containing the pure products were collected, evaporated and dried until constant weight: 88 mg (13%) product **16** and 123 mg (13%) product **17.**

Table S8, entry 3: The 784 mg crude product obtained after lyophilization of the aqueous phase was purified by column chromatography on 10 g silica gel (0.040–0.063 mesh size), with gradient elution: $CHCl_3$, followed by $CHCl_3$/MeOH 5/1, 2/1 and 1/1. The fractions containing the pure products were collected, evaporated and dried until constant weight:

64 mg (7%) white oily solid **1,3-bis(2-hydroxypropyl)pyrimidine-2,4(1*H*,3*H*)-dione (17)**, $C_{10}H_{16}N_2O_4$: 228.25, Rf = 0.55 ($CHCl_3$/MeOH 5/1), rt = 0.22’ (100%), m/z = 229;

209 mg (34%) light yellow solid **2-methyl-2,3-dihydro-[1,3]oxazolo[3,2-a]pyrimidin-7-one (18),** $C_7H_8N_2O_2$: 152.15, CAS Reg. No: 261920-67-6 [12, 13], Rf = 0.13 ($CHCl_3$/MeOH 5/1), Mp. > 250 °C (decomp.), rt = 0.21’ (100%), m/z = 153 and 327 [2M + Na];

^{1}H NMR (400 MHz, DMSO-d_6+D_2O 9:1): δ = 1.40 (d, *J* = 6.2Hz, 3H, CH_3), 3.85 (dd, *J* = 10.3, 7.6Hz, 1H, CH_2), 4.34 (dd, *J* = 11.3, 8.8Hz, 1H, CH_2), 5.12 (m, 1H, OC*H*), 5.94 (d, *J* = 7.4Hz, 1H, O=CCH), 7.59 (d, *J* = 7.4Hz, 1H, NCH).

^{13}C NMR (100 MHz, DMSO-d_6+D_2O): δ = 20.7, 54.2, 79.9, 109.0, 142.2, 163.1, 177.1.

114 mg (17%) yellow solid **2-methyl-2,3-dihydro-[1,3]thiazolo[3,2-a] pyrimidin-7-one (19),** $C_7H_8N_2OS$: 168.21, CAS Reg. No: 907171-18-0, Rf = 0.30 ($CHCl_3$/MeOH 5/1), Mp: 167.6-169.3 °C, rt = 0.21’ (100%), m/z = 169.

^{1}H NMR (400 MHz, DMSO-d_6): δ = 1.44 (d, J = 6.7Hz, 3H, CH_3), 4.04 (dd, J = 11.3, 5.8Hz, 1H, CH_2), 4.12 (m, 1H, SC*H*), 4.42 (dd, J = 11.3, 7.0Hz, 1H, CH_2), 5.84 (d, J = 7.5Hz, 1H, O=CCH), 7.76 (d, J = 7.5Hz, 1H, NCH).
^{13}C NMR (100 MHz, DMSO-d_6): δ = 20.2, 39.5, 59.5, 108.1, 140.8, 166.6, 168.6.
Table S8, entry 4: The 750 mg crude product obtained after lyophilization of the aqueous phase was purified by column chromatography on 10 g silica gel (0.040–0.063 mesh size), with gradient elution: $CHCl_3$/MeOH 5/1, 2/1 and 1/1. The fractions containing the pure products were collected, evaporated and dried until constant weight:
276 mg (30%) white oily solid **1,3-bis(2-hydroxypropyl)pyrimidine-2,4(1*H*,3*H*)-dione (17)**, 123 mg (20%) light yellow solid **2-methyl-2,3-dihydro-[1,3] oxazolo[3,2-a]pyrimidin-7-one (18),** and 91 mg (14%) yellow solid **2-methyl-2,3-dihydro-[1,3]thiazolo[3,2-a] pyrimidin-7-one (19).**

HPLC-MS data, ^{1}H- and ^{13}C-NMR spectra

2-(2-hydroxypropyl)-1*H*-isoindole-1,3(2*H*)-dione (8)

Figure S1. HPLC-MS data of **8**

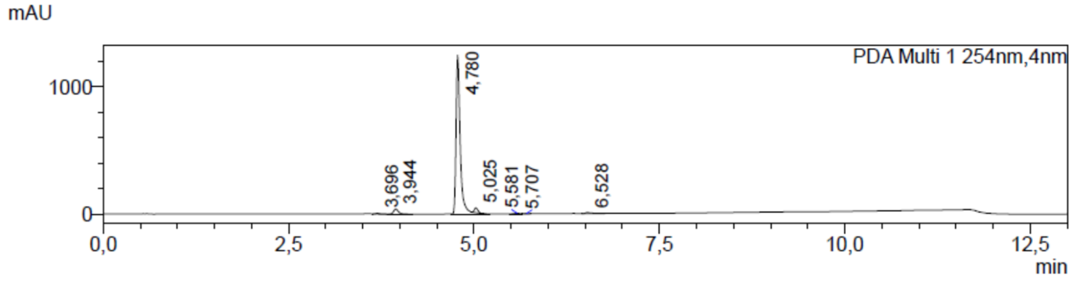

Peak Table

PDA Ch1 254nm

Peak#	Ret. Time	Area	Height	Conc.	Name	Area%	Height%
1	3,696	18751	6018	0,358		0,358	0,444
2	3,944	182946	40794	3,489		3,489	3,012
3	4,780	4837330	1253663	92,255		92,255	92,559
4	5,025	119947	38366	2,288		2,288	2,833
5	5,581	22491	5080	0,429		0,429	0,375
6	5,707	7701	1961	0,147		0,147	0,145
7	6,528	54277	8558	1,035		1,035	0,632
Total		5243443	1354440			100,000	100,000

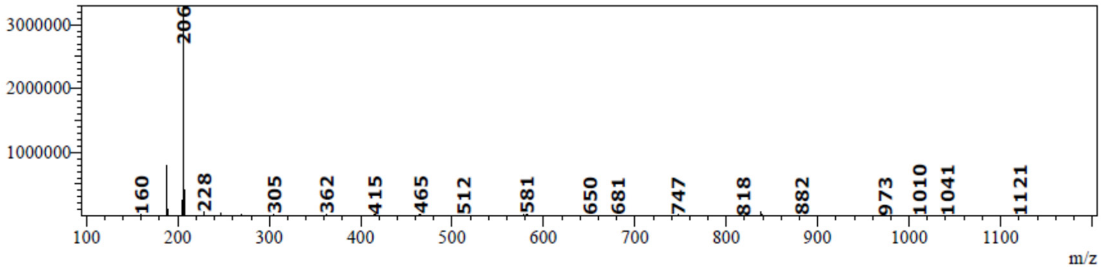

Figure S2. ^{1}H NMR spectrum of **8**

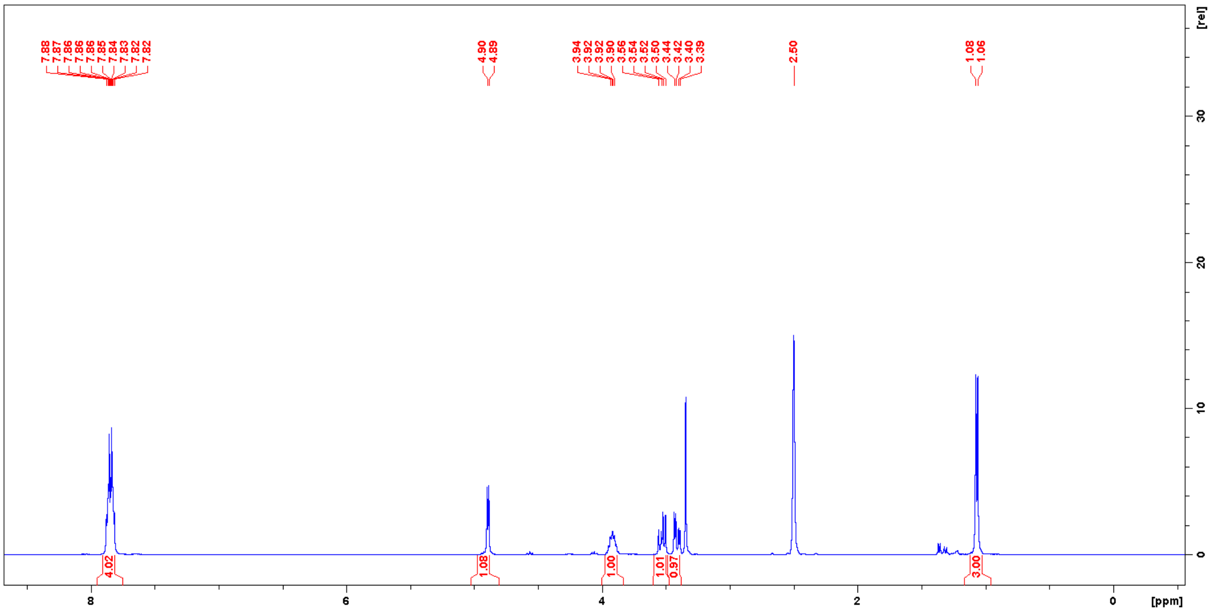

Figure S3. ^{13}C NMR spectrum of **8**

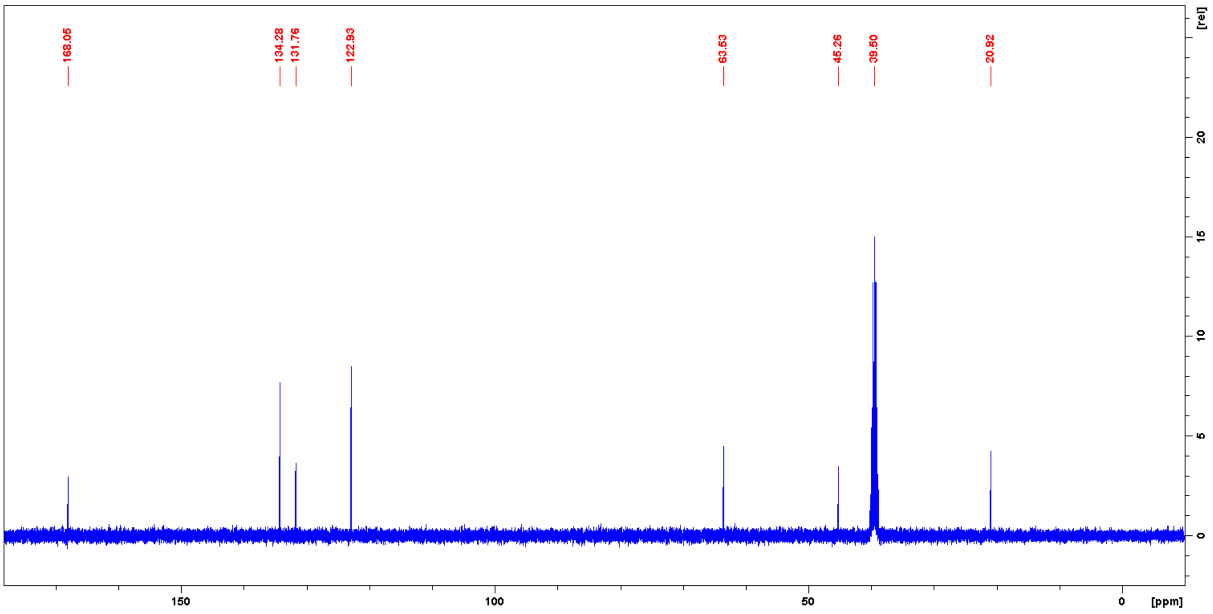

1-(2-hydroxypropyl)-1*H*-indole-2,3-dione (9)

Figure S4. HPLC-MS data of **9**

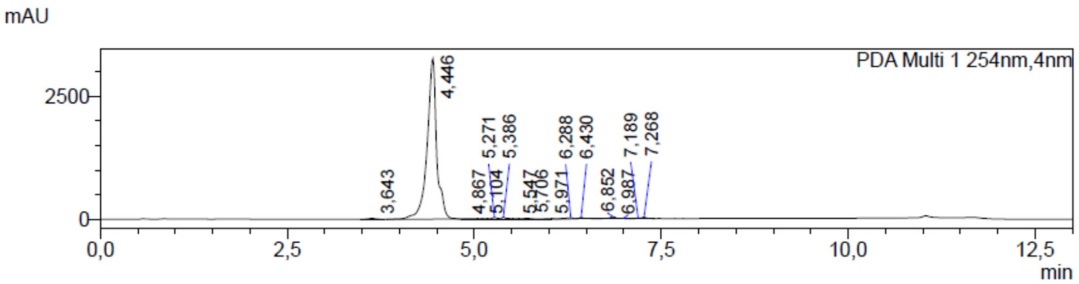

Peak Table

PDA Ch1 254nm

Peak#	Ret. Time	Area	Height	Conc.	Name	Area%	Height%
1	3,643	88987	14115	0,291		0,291	0,399
2	4,446	29089657	3264989	95,218		95,218	92,261
3	4,867	78091	8891	0,256		0,256	0,251
4	5,104	44971	9177	0,147		0,147	0,259
5	5,271	148485	31609	0,486		0,486	0,893
6	5,386	174038	36695	0,570		0,570	1,037
7	5,547	24635	6462	0,081		0,081	0,183
8	5,706	89163	14292	0,292		0,292	0,404
9	5,971	21662	6041	0,071		0,071	0,171
10	6,288	180584	22339	0,591		0,591	0,631
11	6,430	156506	29581	0,512		0,512	0,836
12	6,852	257520	44207	0,843		0,843	1,249
13	6,987	17234	4423	0,056		0,056	0,125
14	7,189	58659	17055	0,192		0,192	0,482
15	7,268	120538	28993	0,395		0,395	0,819
Total		30550730	3538869			100,000	100,000

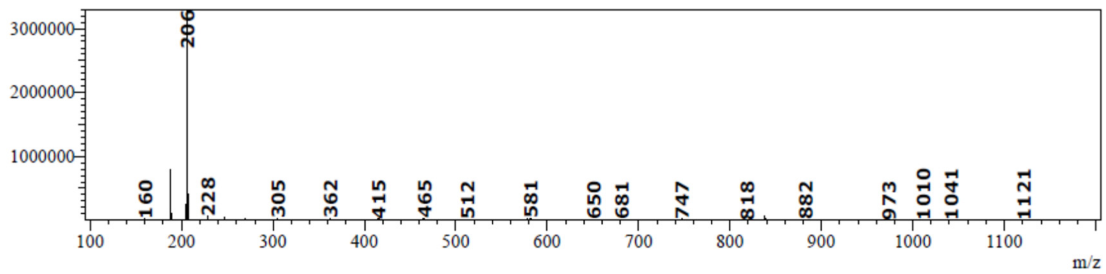

Figure S5. ^{1}H NMR spectrum of **9**

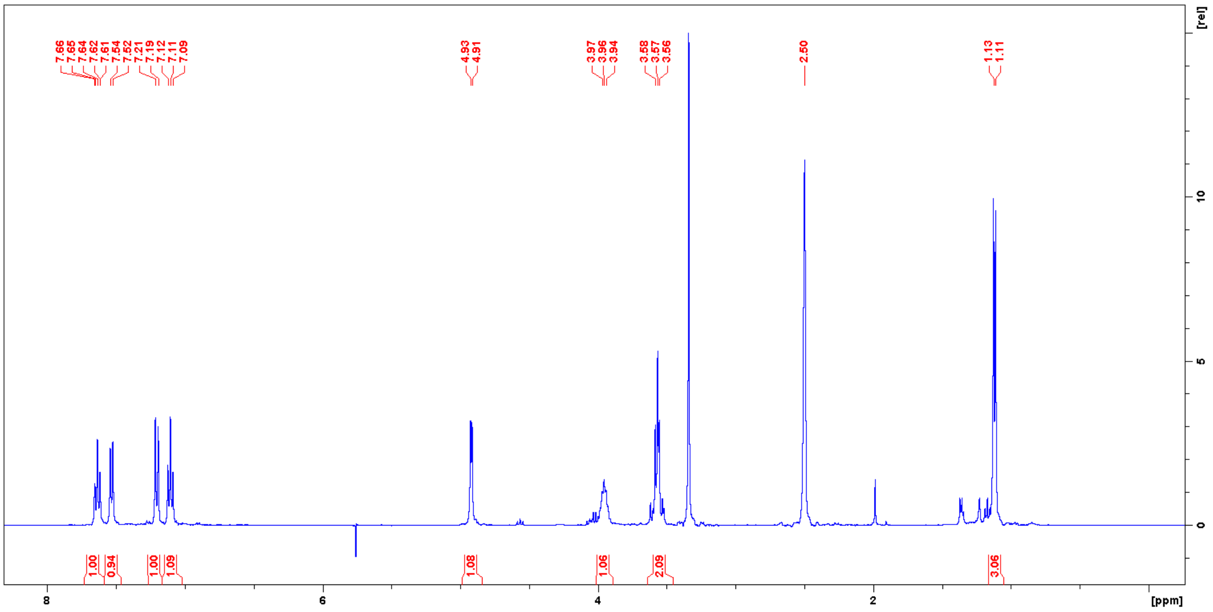

Figure S6. ^{13}C NMR spectrum of **9**

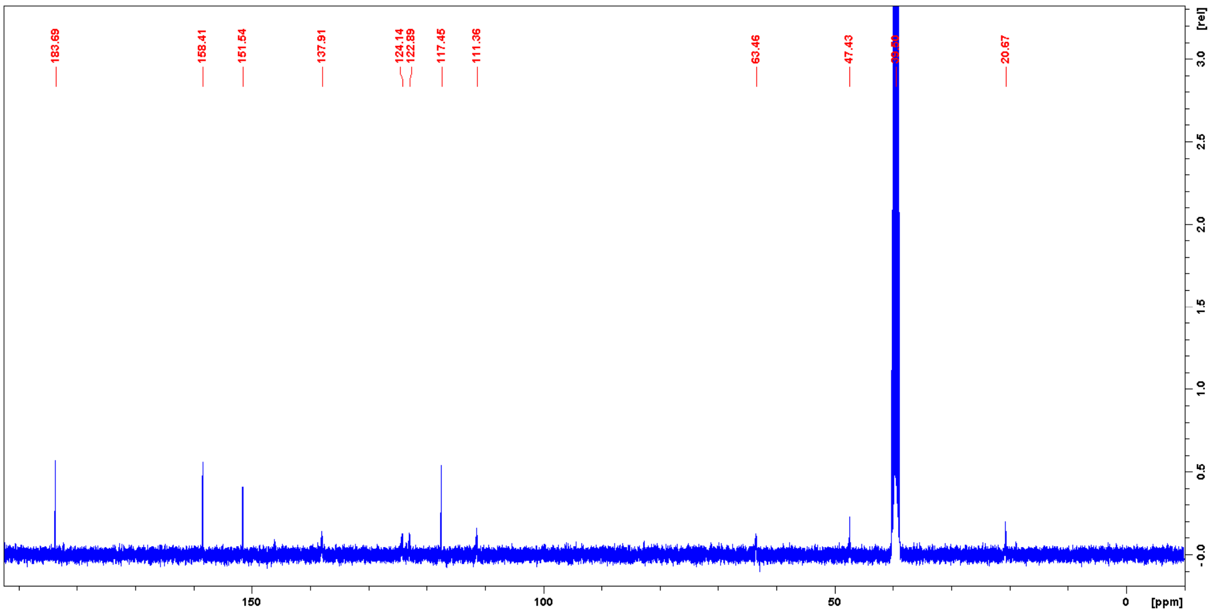

2-(2-hydroxypropyl)phthalazin-1(2*H*)-one (10)

Figure S7. HPLC-MS data of **10**

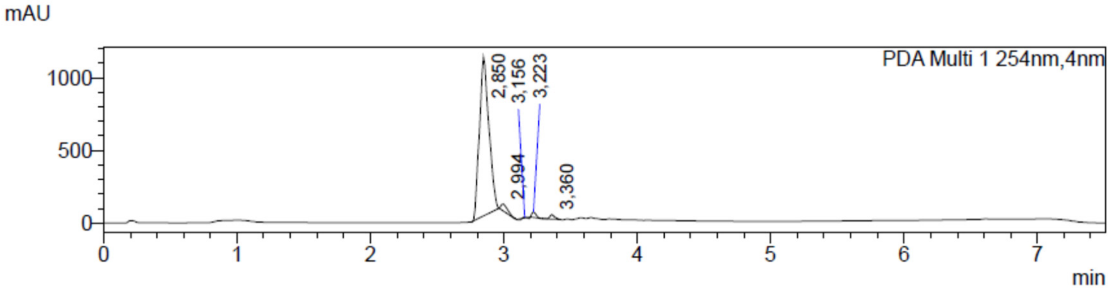

Peak Table

PDA Ch1 254nm

Peak#	Ret. Time	Area	Height	Conc.	Name	Area%	Height%
1	2,850	5435423	1091788	0,000		94,996	89,801
2	2,994	137163	46250	0,000		2,397	3,804
3	3,156	-3071	8025	0,000		-0,054	0,660
4	3,223	77741	39365	0,000		1,359	3,238
5	3,360	74466	30363	0,000		1,301	2,497
Total		5721721	1215791			100,000	100,000

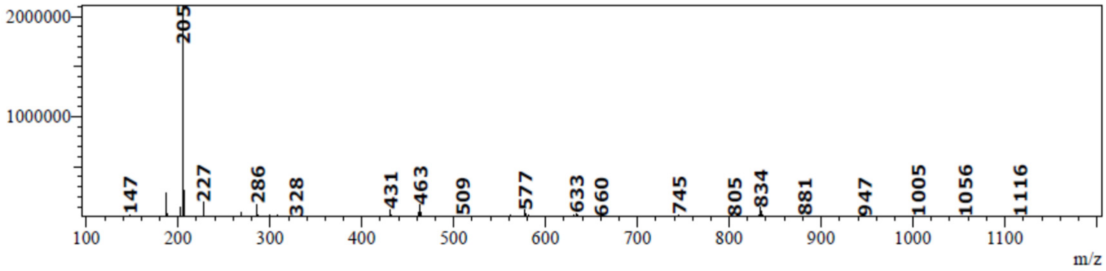

Figure S8. ^{1}H NMR spectrum of **10**

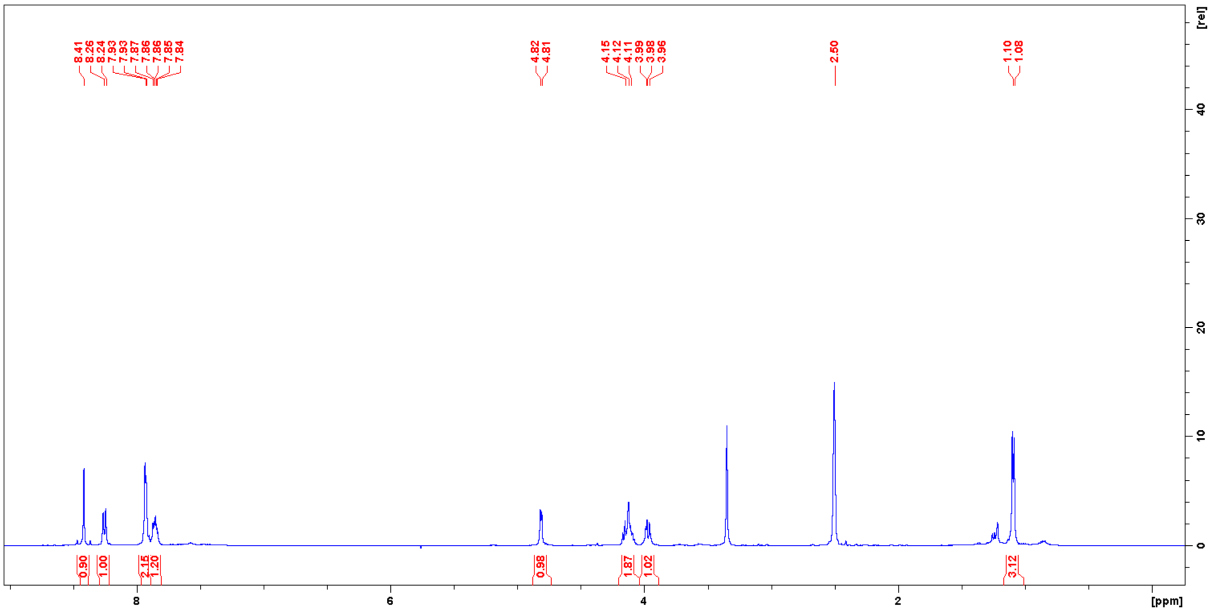

Figure S9. ^{13}C NMR spectrum of **10**

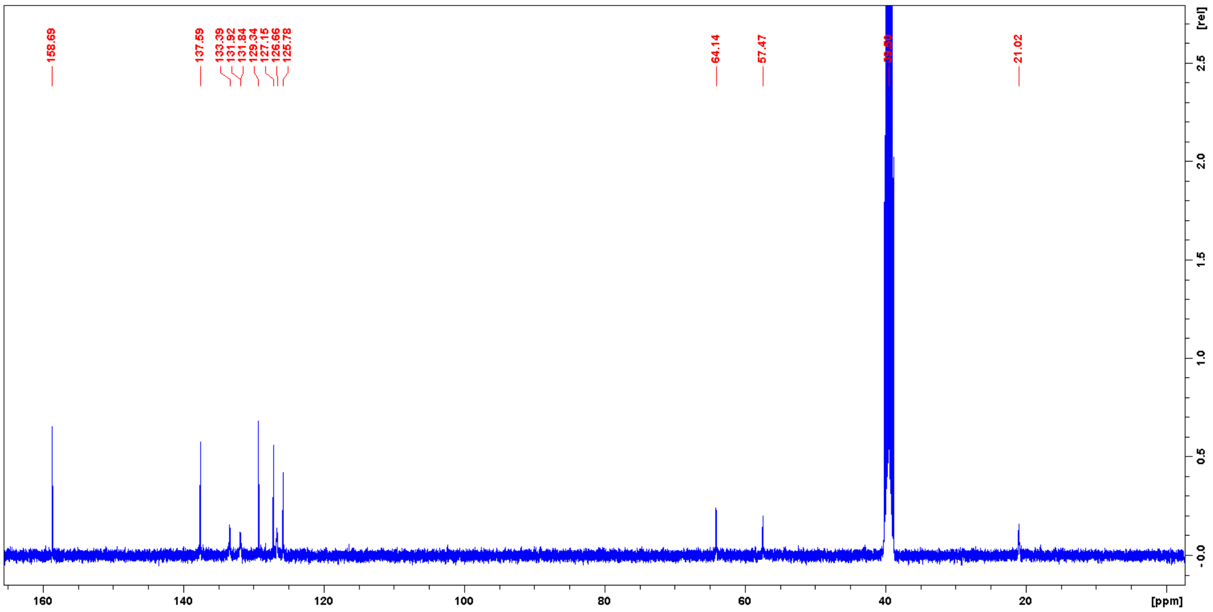

3-(2-hydroxypropyl)pyrimidin-4(3*H*)-one (11)

Figure S10. HPLC-MS data of **11**

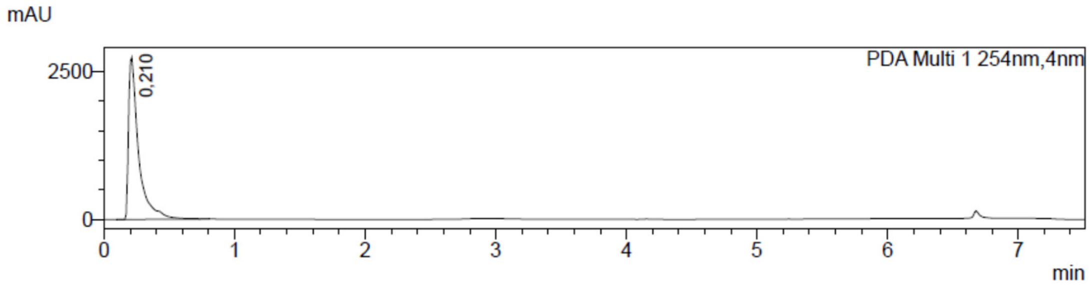

Peak Table

PDA Ch1 254nm

Peak#	Ret. Time	Area	Height	Conc.	Name	Area%	Height%
1	0.210	14623646	2732183	0.000		100.000	100.000
Total		14623646	2732183			100.000	100.000

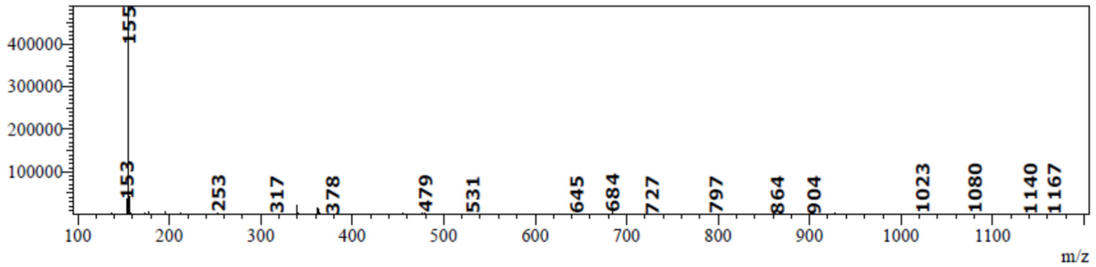

Figure S11. ^{1}H NMR spectrum of **11**

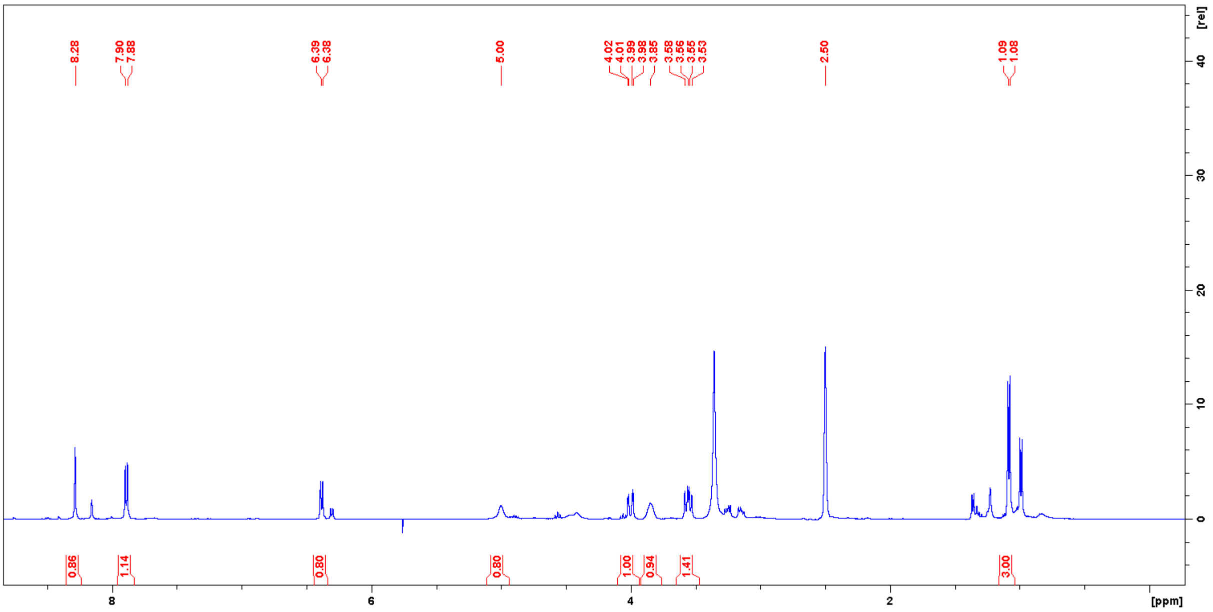

Figure S12. ^{13}C NMR spectrum of **11**

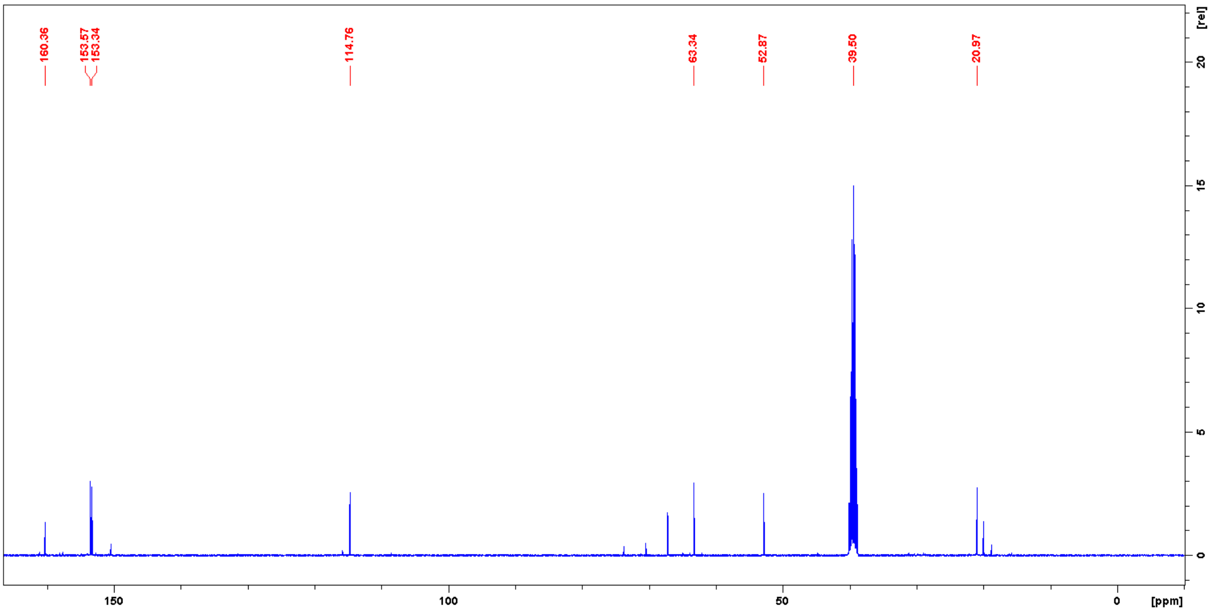

1-(2-hydroxypropyl)pyrimidin-4(1*H*)-one (12)

Figure S13. HPLC-MS data of **12**

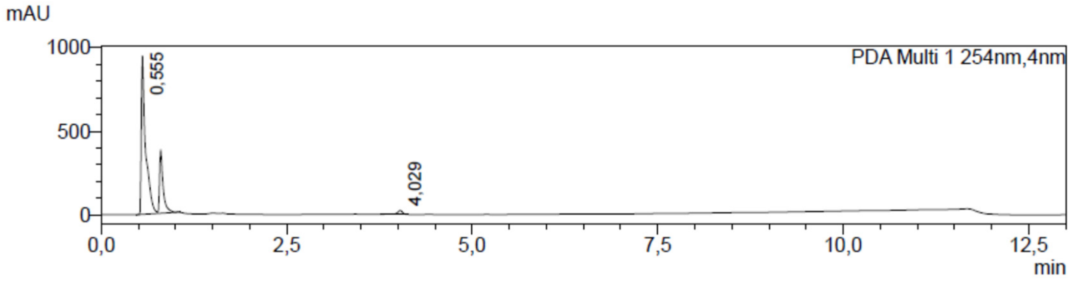

Peak Table

PDA Ch1 254nm

Peak#	Ret. Time	Area	Height	Conc.	Name	Area%	Height%
1	0,555	5088055	951036	0,000		97,438	97,470
2	4,029	133807	24689	0,000		2,562	2,530
Total		5221862	975725			100,000	100,000

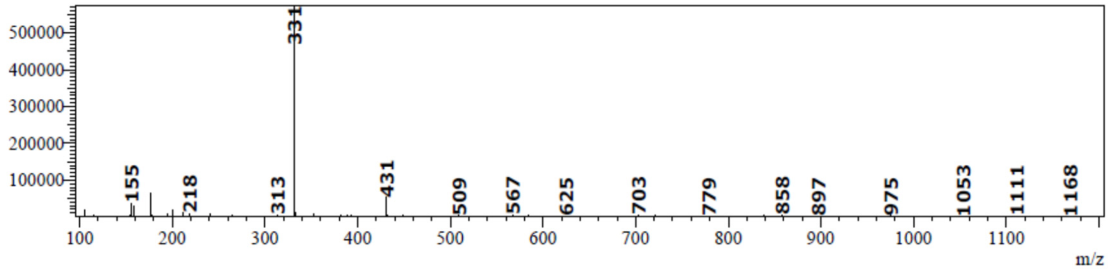

Figure S14. ^{1}H NMR spectrum of **12**

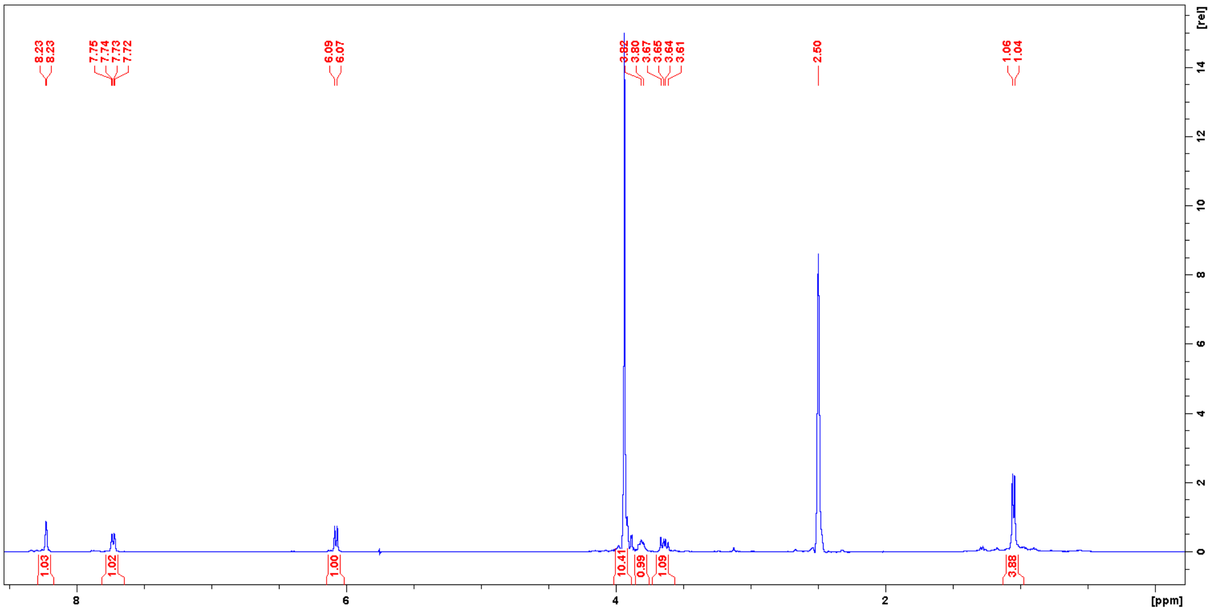

Figure S15. ^{13}C NMR spectrum of **12**

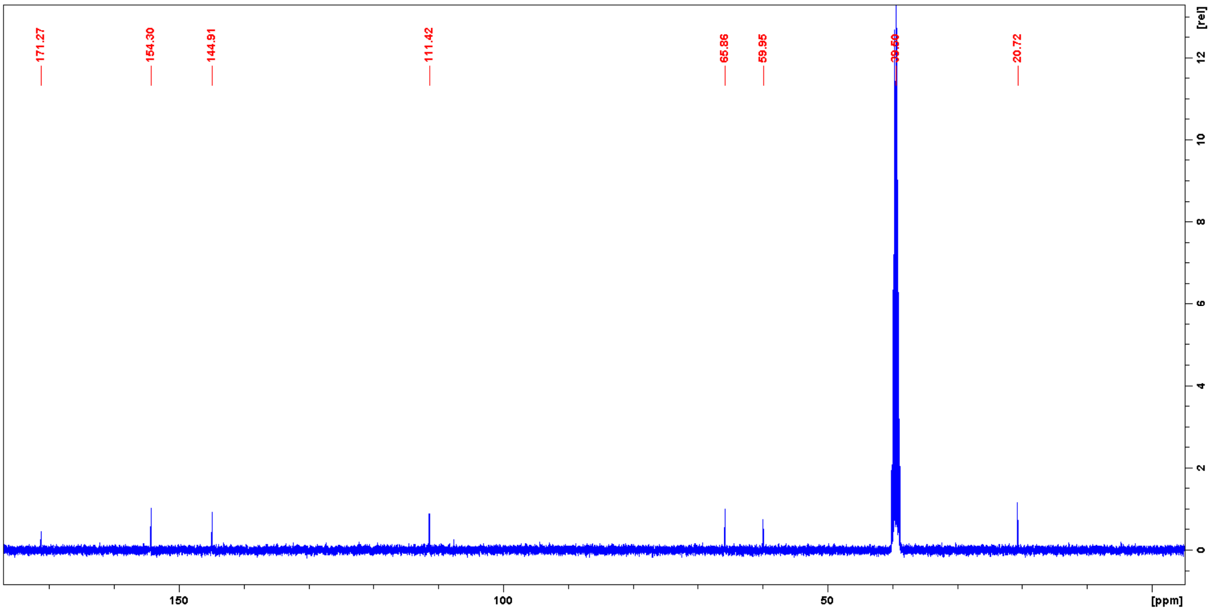

1,3-bis(2-hydroxypropyl)-6-methylpyrimidine-2,4(1*H*,3*H*)-dione (13)

Figure S16. HPLC-MS data of **13**

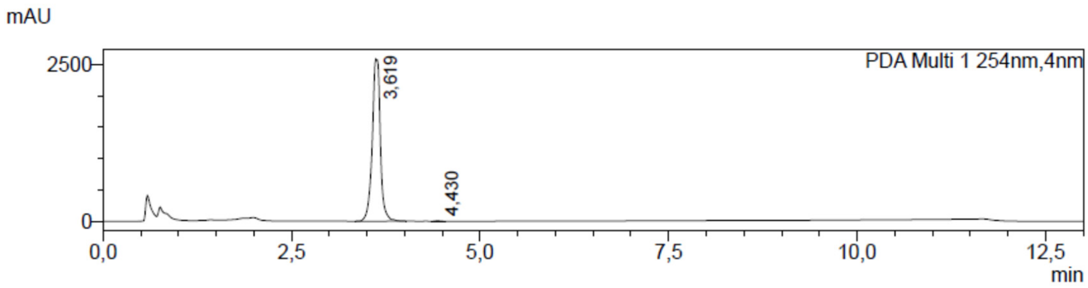

Peak Table

PDA Ch1 254nm

Peak#	Ret. Time	Area	Height	Conc.	Name	Area%	Height%
1	3,619	19305763	2588217	99,825		99,825	99,657
2	4,430	33865	8916	0,175		0,175	0,343
Total		19339628	2597133			100,000	100,000

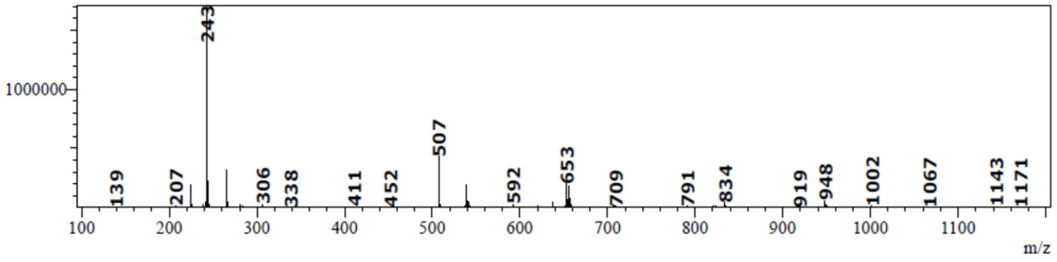

Figure S17. ^{1}H NMR spectrum of **13**

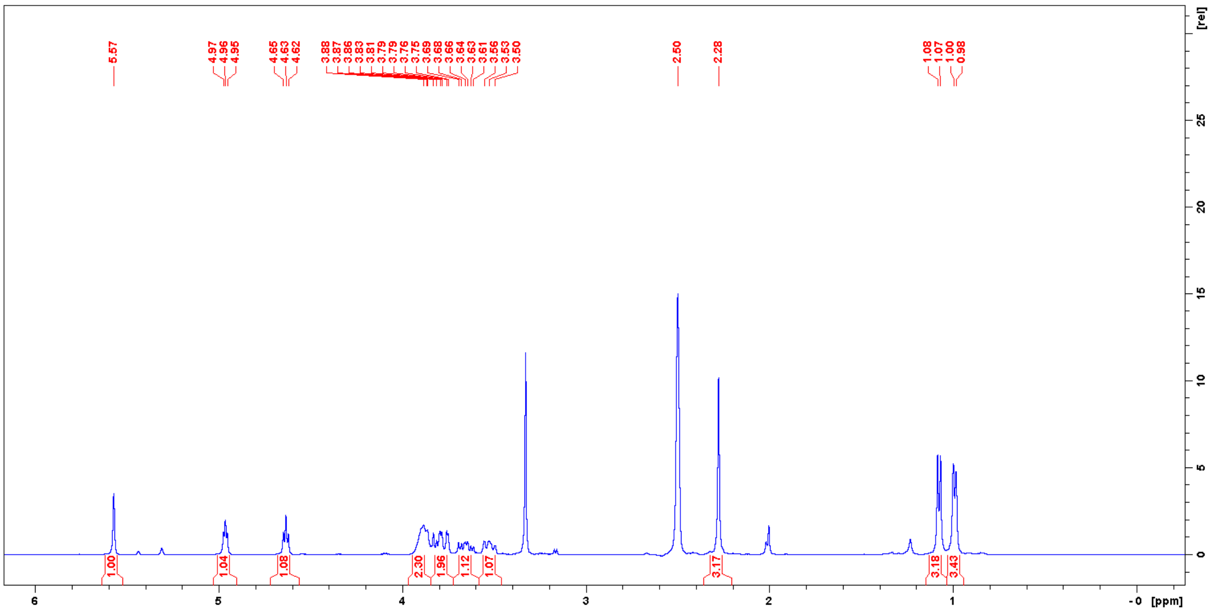

Figure S18. ^{13}C NMR spectrum of **13**

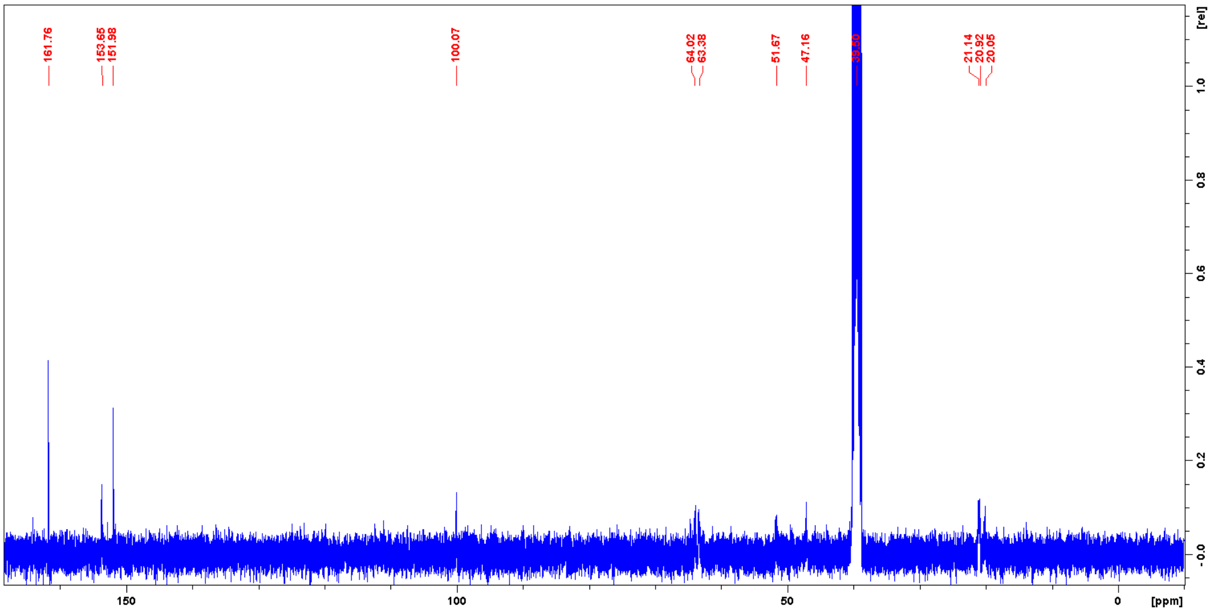

2-(2-hydroxypropyl)benzotriazole (14)

Figure S19. HPLC-MS data of **14**

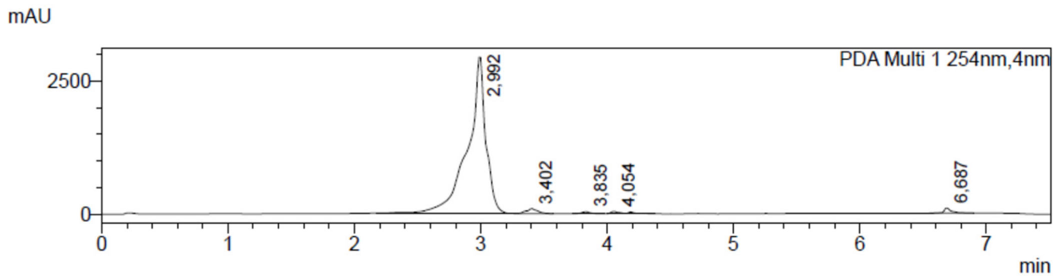

Peak Table

PDA Ch1 254nm

Peak#	Ret. Time	Area	Height	Conc.	Area%	Height%
1	2,992	28763981	2920061	0,000	95,095	91,414
2	3,402	586801	95346	0,000	1,940	2,985
3	3,835	183930	40290	0,000	0,608	1,261
4	4,054	337963	41915	0,000	1,117	1,312
5	6,687	374809	96704	0,000	1,239	3,027
Total		30247484	3194316		100,000	100,000

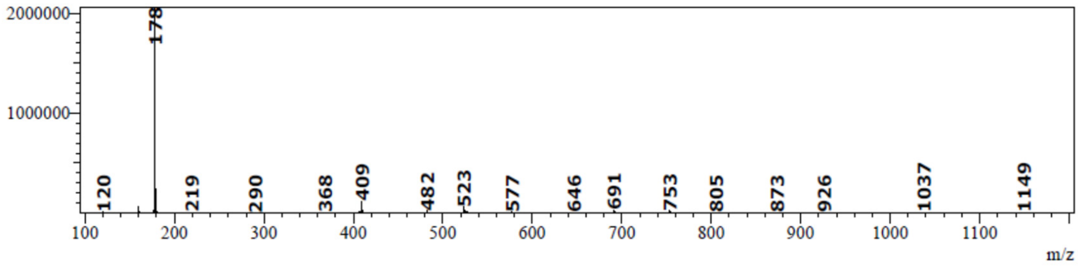

Figure S20. ^{1}H NMR spectrum of **14**

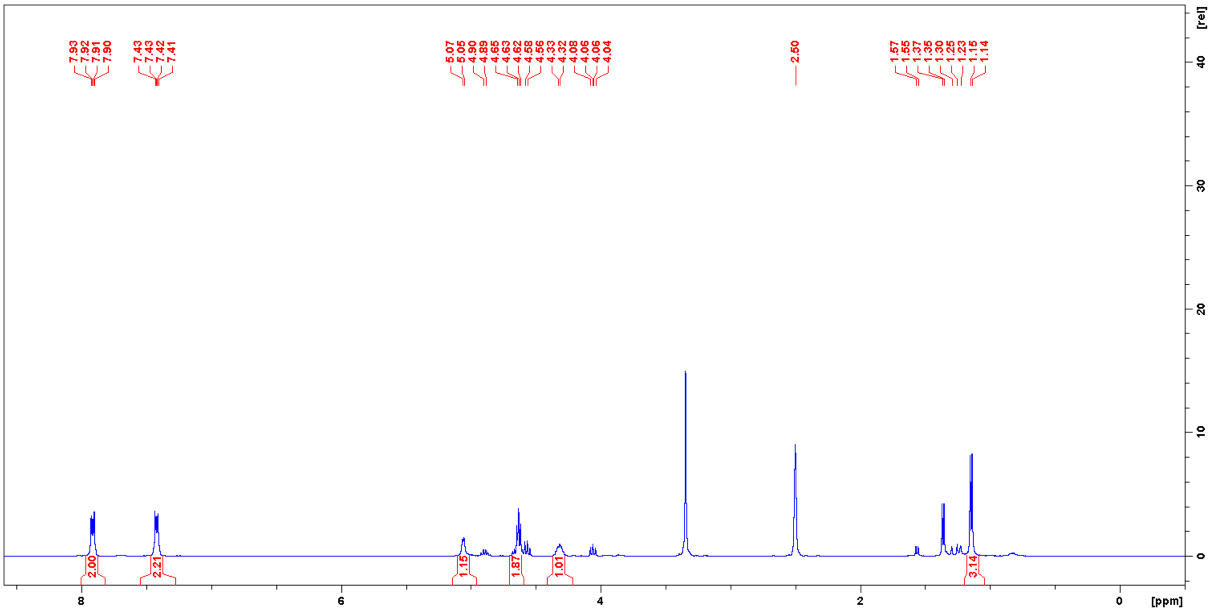

Figure S21. ^{13}C NMR spectrum of **14**

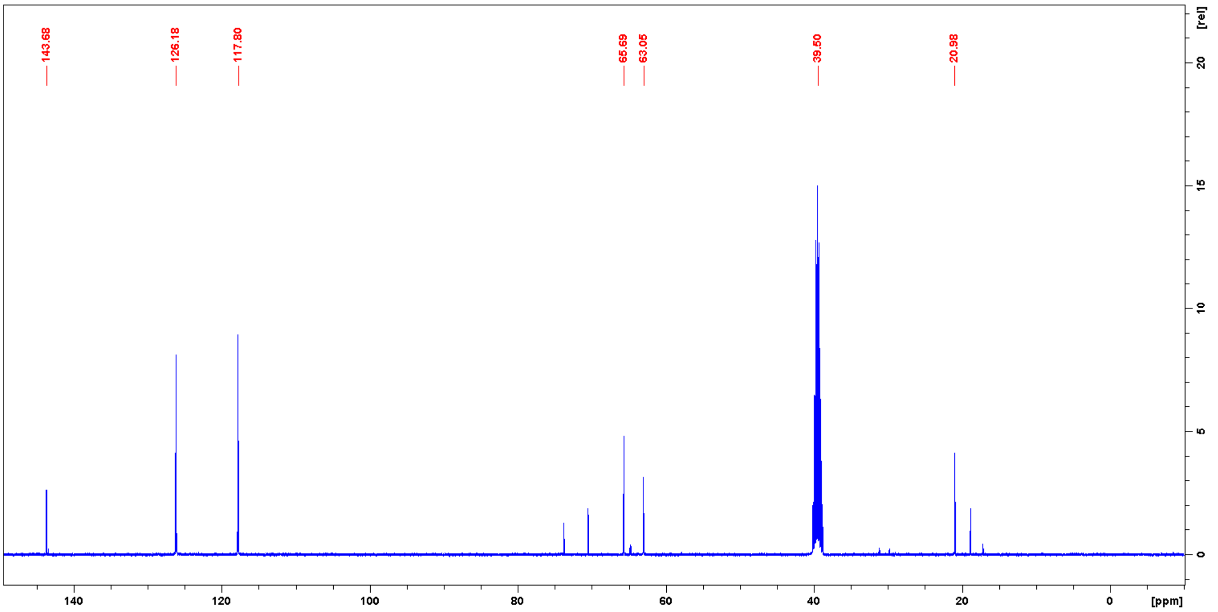

1-(2-hydroxypropyl) benzotriazole (15)

Figure S22. HPLC-MS data of **15**

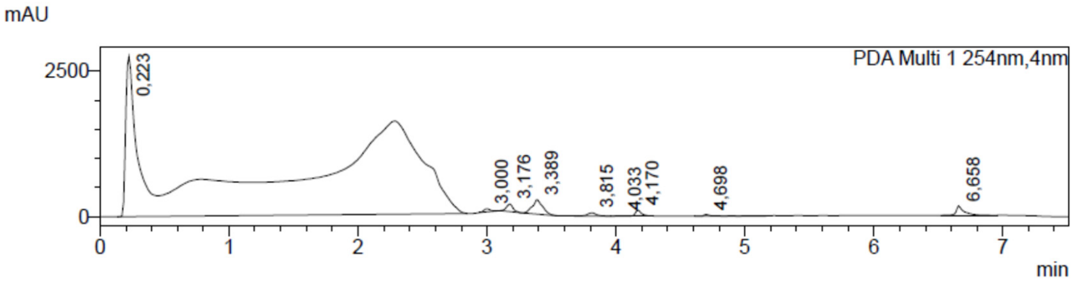

Peak Table

PDA Ch1 254nm

Peak#	Ret. Time	Area	Height	Conc.	Area%	Height%
1	0,223	120562288	2751088	0,000	97,297	77,911
2	3,000	200163	53982	0,000	0,162	1,529
3	3,176	439607	130072	0,000	0,355	3,684
4	3,389	1353830	245208	0,000	1,093	6,944
5	3,815	255305	52707	0,000	0,206	1,493
6	4,033	26079	7480	0,000	0,021	0,212
7	4,170	312897	100214	0,000	0,253	2,838
8	4,698	96849	27419	0,000	0,078	0,777
9	6,658	664787	162877	0,000	0,537	4,613
Total		123911806	3531047		100,000	100,000

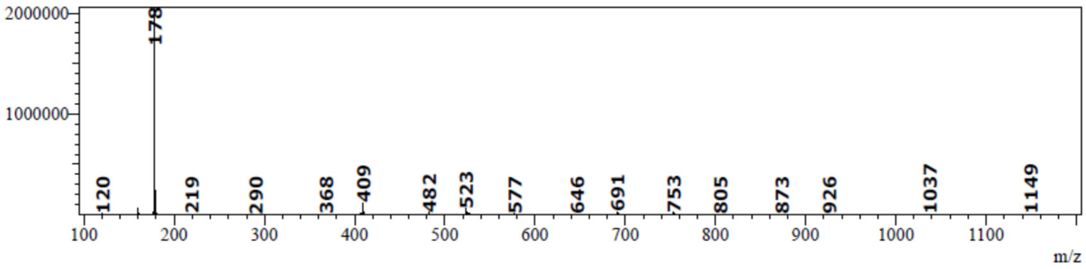

Figure S23. ^{1}H NMR spectrum of **15**

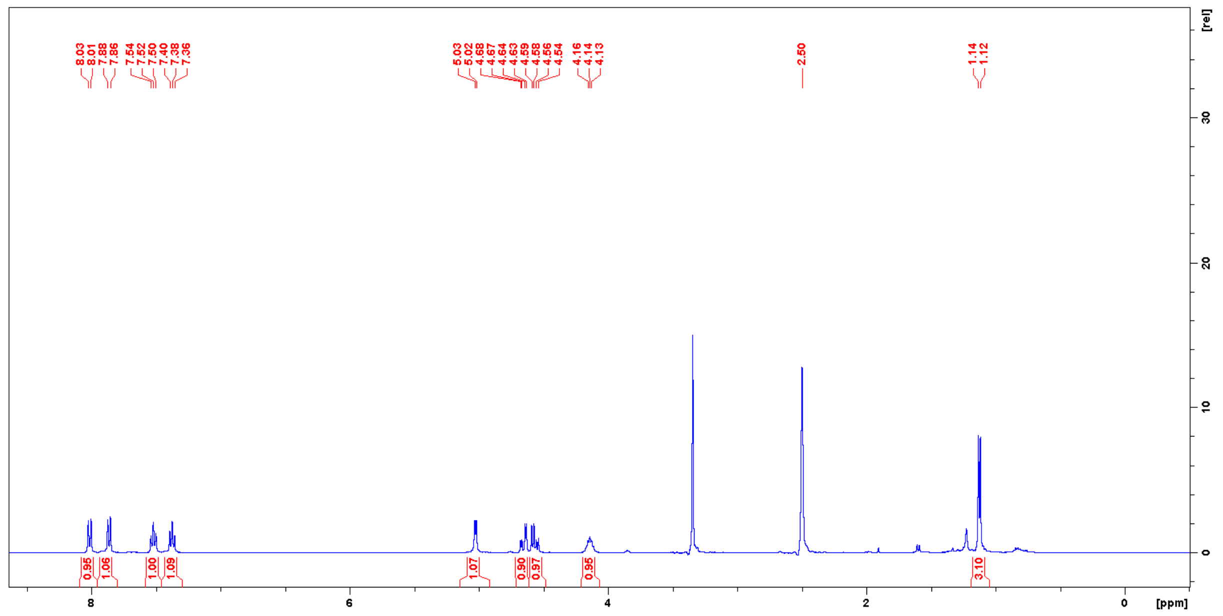

Figure S24. ^{13}C NMR spectrum of **15**

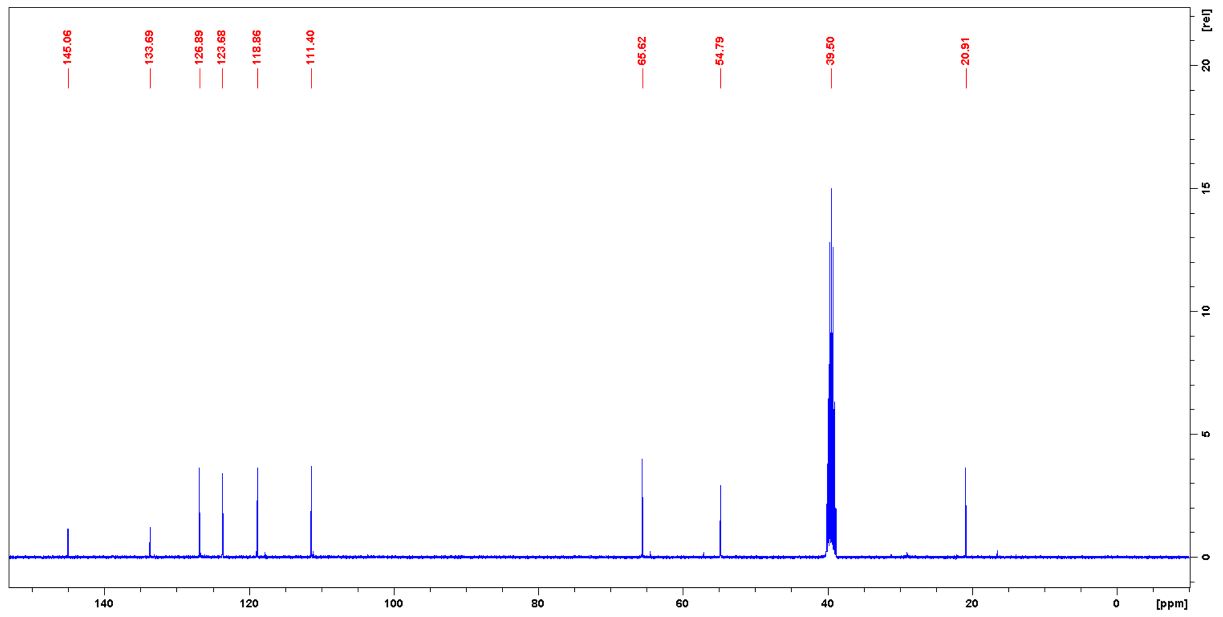

1-(2-hydroxypropyl)pyrimidine-2,4(1*H*,3*H*)-dione (16)

Figure S25. HPLC-MS data of **16**

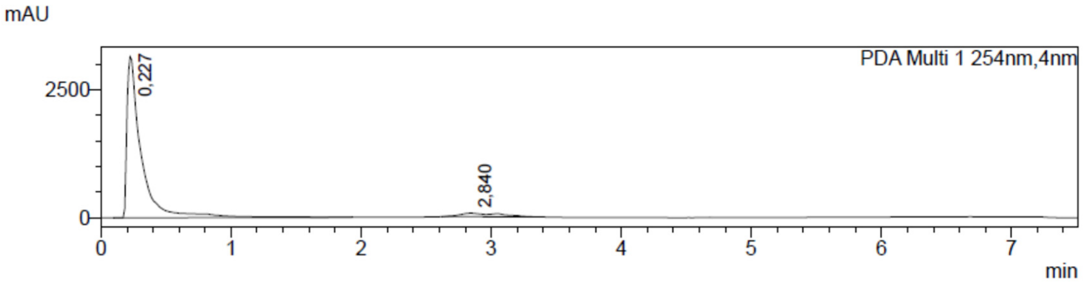

Peak Table

PDA Ch1 254nm

Peak#	Ret. Time	Area	Height	Conc.	Name	Area%	Height%
1	0,227	24193601	3153365	0,000		94,259	97,855
2	2,840	1473566	69136	0,000		5,741	2,145
Total		25667167	3222501			100,000	100,000

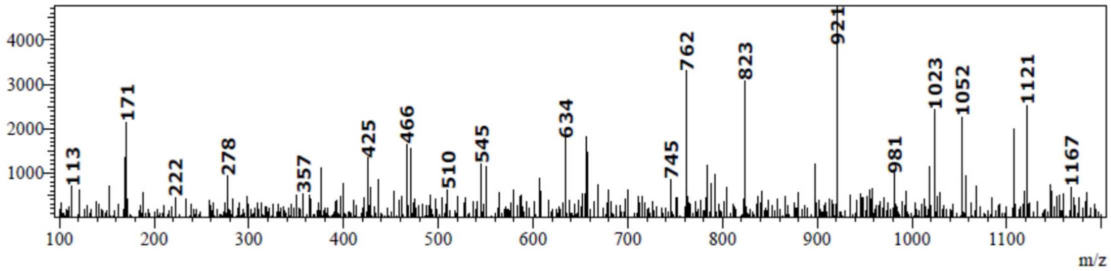

Figure S26. ^{1}H NMR spectrum of **16**

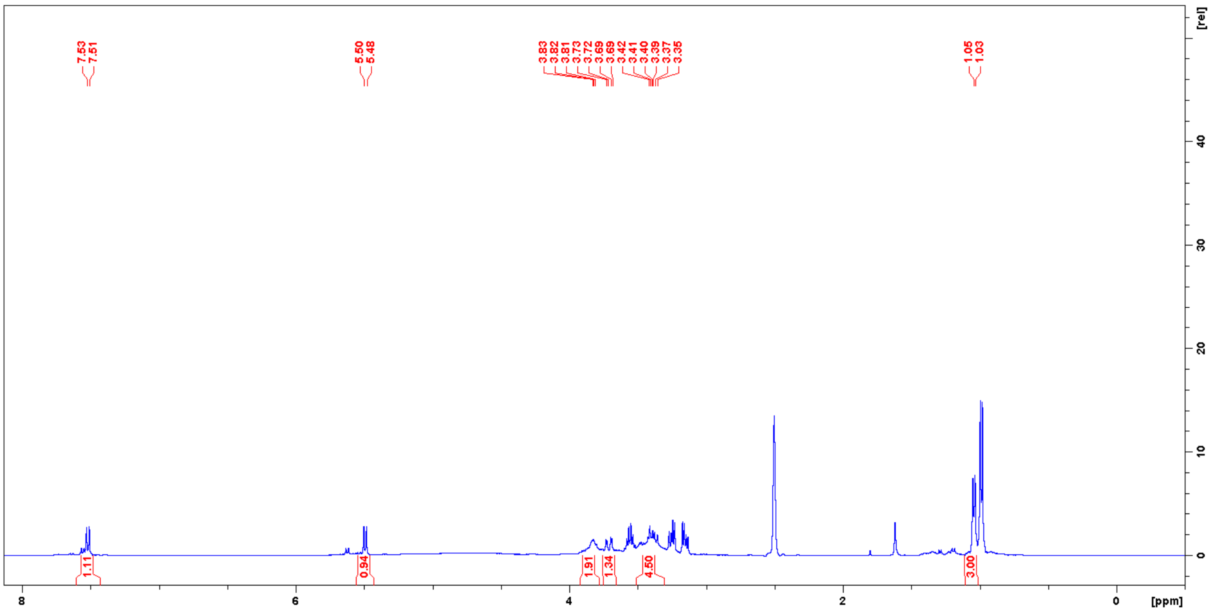

Figure S27. ^{13}C NMR spectrum of **16**

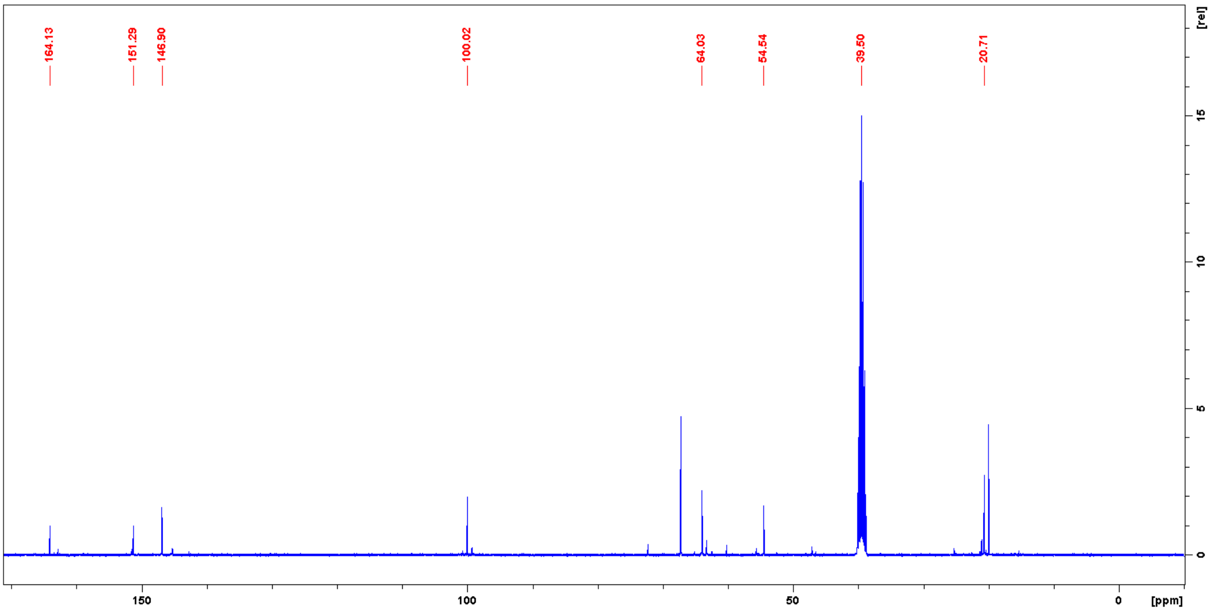

1,3-bis(2-hydroxypropyl)pyrimidine-2,4(1*H*,3*H*)-dione (17)

Figure S28. HPLC-MS data of **17**

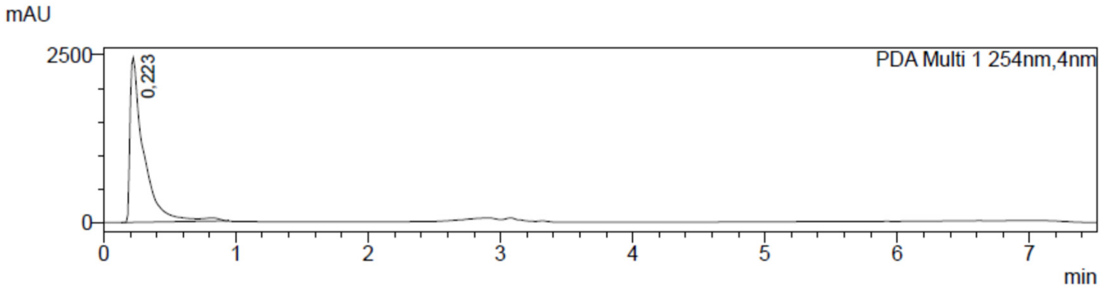

Peak Table

PDA Ch1 254nm

Peak#	Ret. Time	Area	Height	Conc.	Name	Area%	Height%
1	0,223	17773135	2458250	100,000		100,000	100,000
Total		17773135	2458250			100,000	100,000

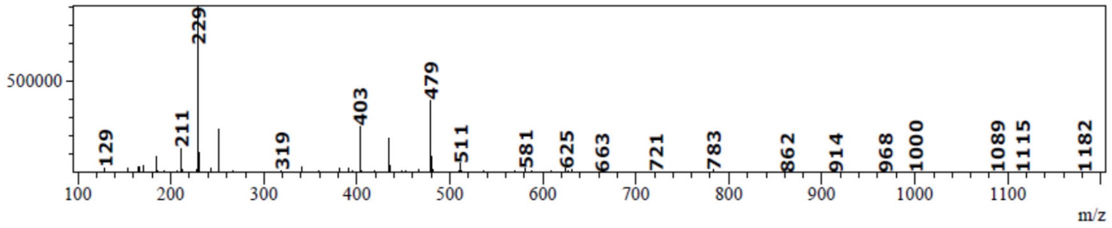

Figure S29. ^{1}H NMR spectrum of **17**

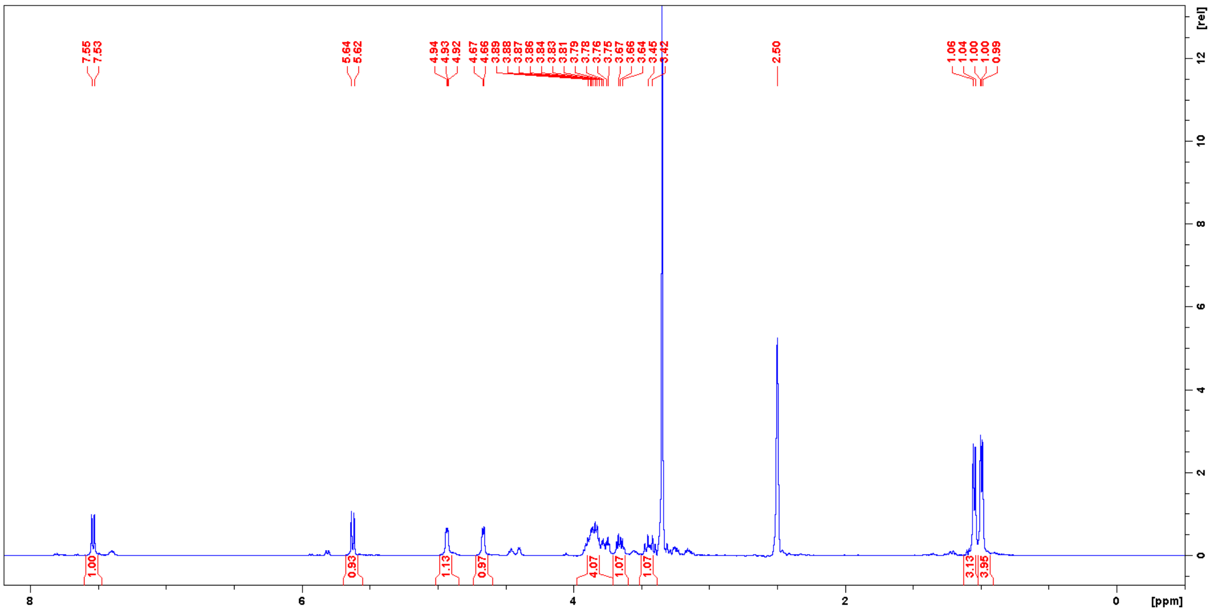

Figure S30. ^{13}C NMR spectrum of **17**

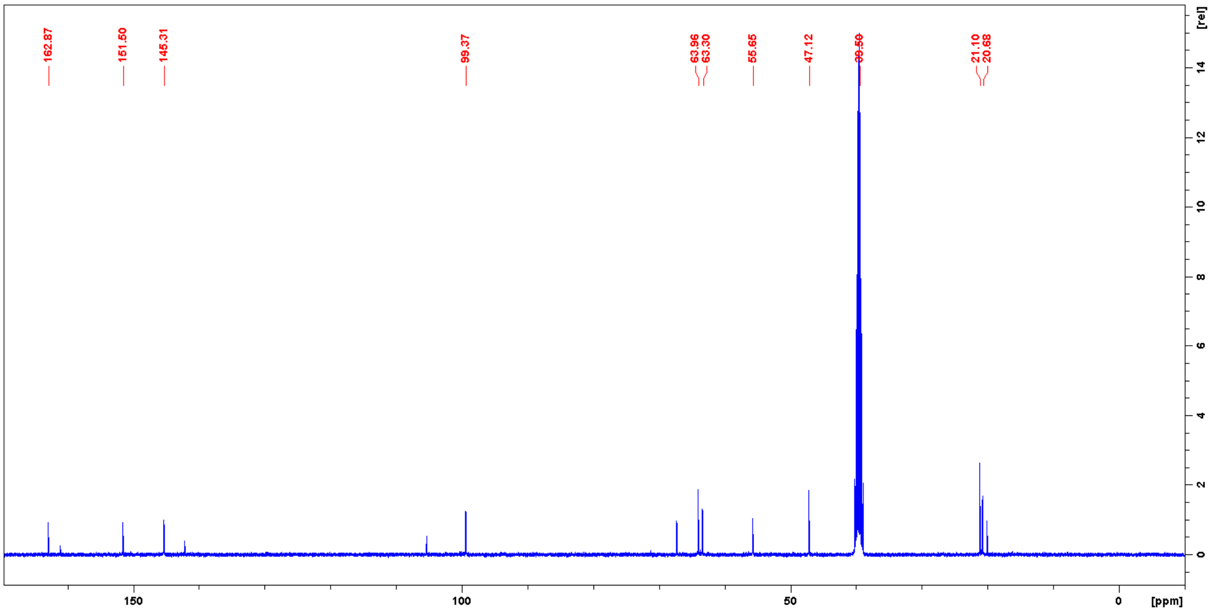

2-methyl-2,3-dihydro-[1,3]oxazolo[3,2-a]pyrimidin-7-one (18)

Figure S31. HPLC-MS data of **18**

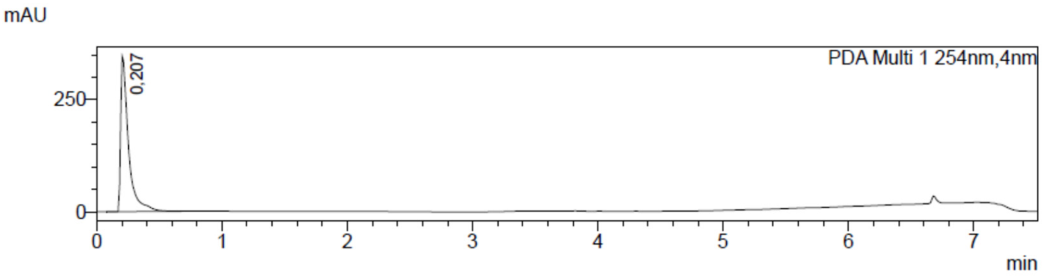

Peak Table

PDA Ch1 254nm

Peak#	Ret. Time	Area	Height	Conc.	Name	Area%	Height%
1	0.207	1504280	347441	0.000		100.000	100.000
Total		1504280	347441			100.000	100.000

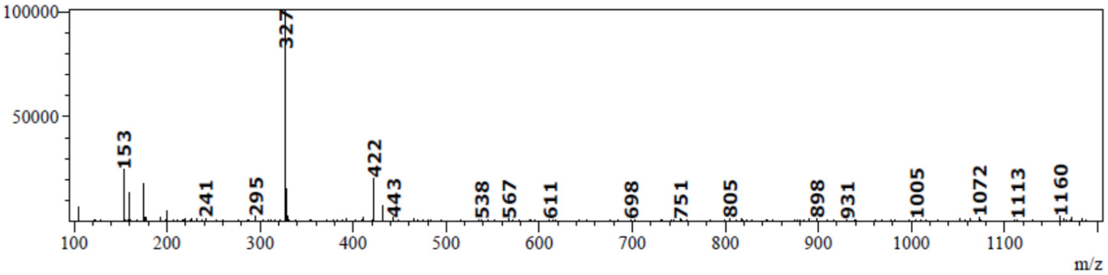

Figure S32. ^{1}H NMR spectrum of **18**

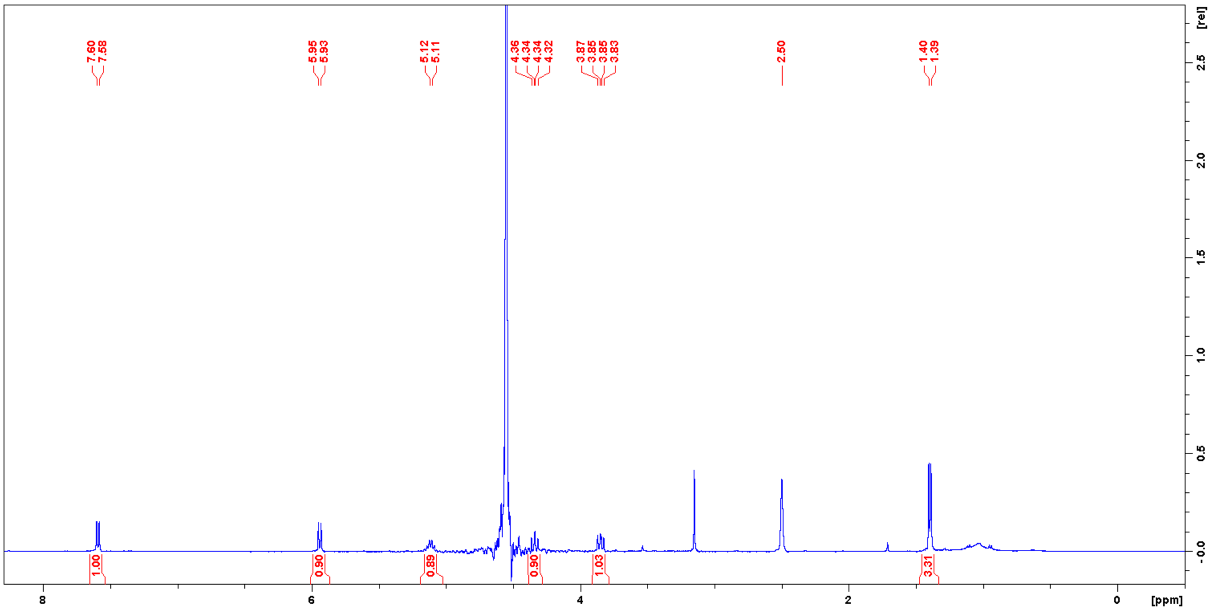

Figure S33. ^{13}C NMR spectrum of **18**

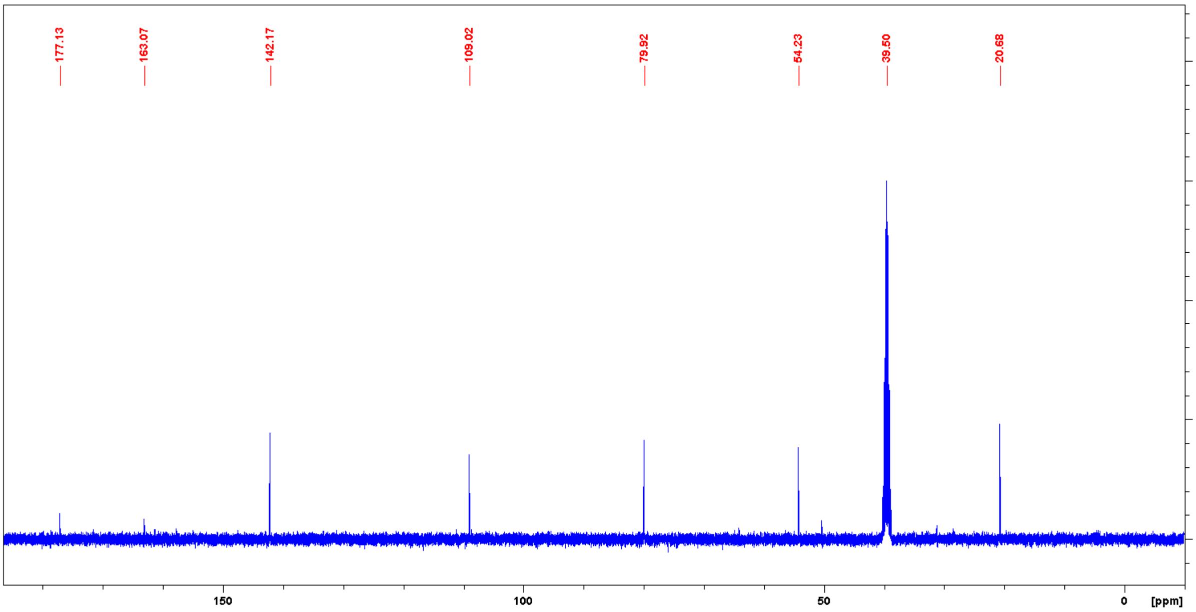

2-methyl-2,3-dihydro-[1,3]thiazolo[3,2-a]pyrimidin-7-one (19)

Figure S34. HPLC-MS data of **19**

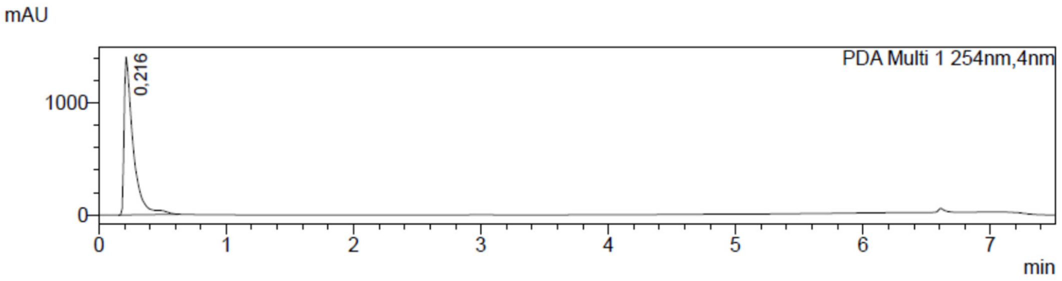

Peak Table

PDA Ch1 254nm

Peak#	Ret. Time	Area	Height	Conc.	Name	Area%	Height%
1	0.216	6974652	1415962	0.000		100.000	100.000
Total		6974652	1415962			100.000	100.000

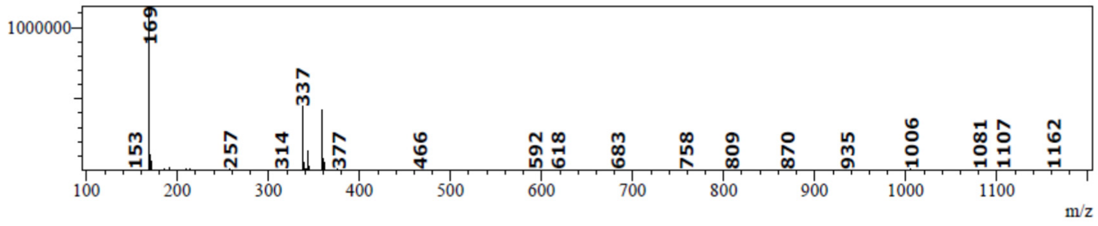

Figure S35. ^{1}H NMR spectrum of **19**

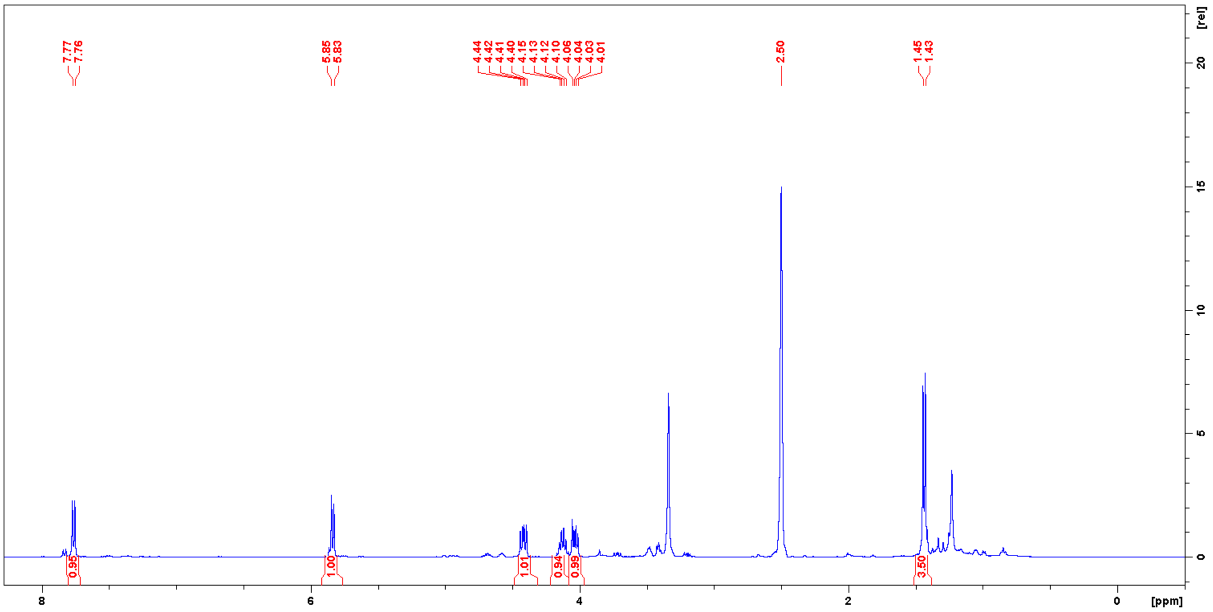

Figure S36. ^{13}C NMR spectrum of **19**

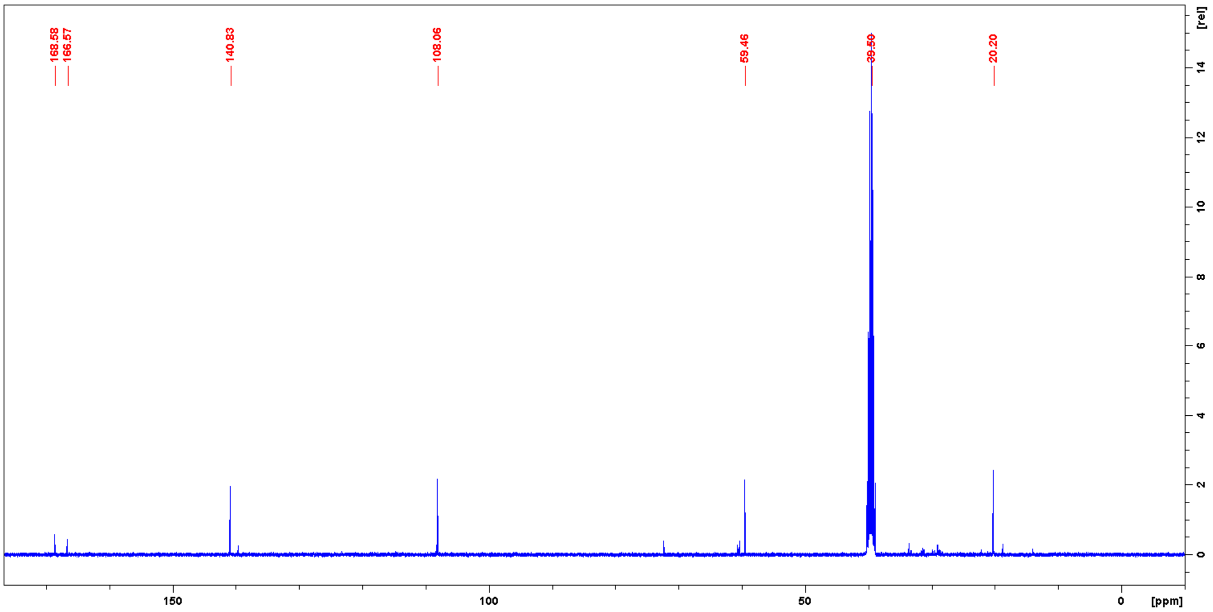

Theoretical calculations

The Jaguar pKa prediction module calculates the pKa (or pKb) of molecules that contain acidic or basic functional groups (Table S9). The calculations involve geometry optimization of the ionic and neutral species, single point en. and frequency calculations, and an empirical correction. The conformational searches were completed with Schrödinger's MacroModel package. The Jaguar pKa module completes the geometry optimization on a DFT (density function theory) level with B3KYP basis set with implicit (water) solvation model6 [14, 15, 16].

MacroModel

Schrödinger Release 2022-1: MacroModel, Schrödinger, LLC, New York, NY, 2021.

Jaguar pKa

Schrödinger Release 2022-1: Jaguar pKa, Schrödinger, LLC, New York, NY, 2021.

Table S9. Schrödinger Jaguar pKa calculations based on the lowest energy conformer (external conformational search)

Structures	lowest en. protonated conformer	lowest en. deprotonated conformer
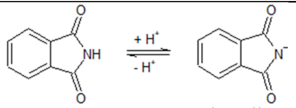 phtalazine pK_a = 8.63 (8.63*)	gas phase en.: -513.117847<br>sol phase en.: -513.138044<br>(hartrees)	gas phase en.: -512.560480<br>sol phase en.: -512.668156<br>(hartrees)
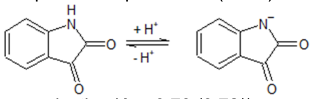 isatin pK_a = 9.70 (9.72*)	gas phase en.: -513.082193<br>sol phase en.: -513.104077<br>(hartrees)	gas phase en.: -512.527494<br>sol phase en.: -512.630304<br>(hartrees)
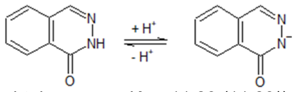 phtalazynone pK_a = 11.89 (11.89*)	gas phase en.: -493.209912<br>sol phase en.: -493.227139<br>(hartrees)	gas phase en.: -492.635548<br>sol phase en.: -492.748877<br>(hartrees)
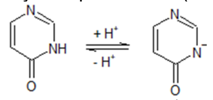 pyrimidone pK_a = 8.40 (8.42*)	gas phase en.: -339.579975<br>sol phase en.: -339.022864<br>(hartrees)	gas phase en.: -339.601320<br>sol phase en.: -339.134750<br>(hartrees)
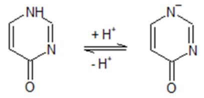 pyrimidone2 pK_a = 7.91 (7.96*)	gas phase en.: -339.555634<br>sol phase en.: -339.584831<br>(hartrees)	gas phase en.: -339.008534<br>sol phase en.: -339.116348<br>(hartrees)
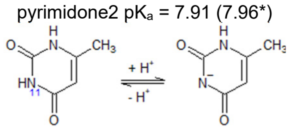 6-methyluracyl (H11) pK_a = 9.34 (9.45*)	gas phase en.: -454.168677<br>sol phase en.: -454.198229<br>(hartrees)	gas phase en.: -453.598637<br>sol phase en.: -453.726849<br>(hartrees)
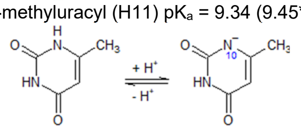 6-methyluracyl (H10) pK_a = 9.44 (9.45*)	gas phase en.: -454.160682<br>sol phase en.: -454.186964<br>(hartrees)	gas phase en.: -453.607240<br>sol phase en.: -453.715793<br>(hartrees)
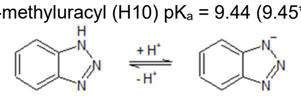 benzotriazole pK_a = 8.00 (7.95*)	gas phase en.: -395.880371<br>sol phase en.: -395.895304<br>(hartrees)	gas phase en.: -395.323155<br>sol phase en.: -395.429454<br>(hartrees)
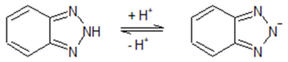 benzotriazole2 pK_a = 6.39 (6.38*)	gas phase en.: -395.881957<br>sol phase en.: -395.896580<br>(hartrees)	gas phase en.: -395.324863<br>sol phase en.: -395.431846<br>(hartrees)

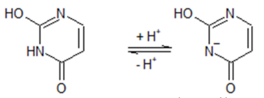<br>uracil pK_a = 8.06 (8.06*)	gas phase en.: -414.823452<br>sol phase en.: -414.848088<br>(hartrees)	gas phase en.: -414.269009<br>sol phase en.: -414.384350<br>(hartrees)
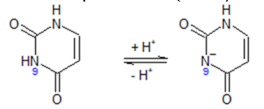<br>uracil2 pK_a (H9) = 9.13 (9.11*)	gas phase en.: -414.842691<br>sol phase en.: -414.872028<br>(hartrees)	gas phase en.: -414.274873<br>sol phase en.: -414.401717<br>(hartrees)
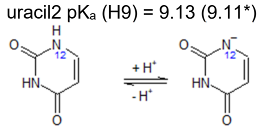<br>uracil2 pK_a (H12) = 8.99 (9.05*)	gas phase en.: -414.834944<br>sol phase en.: -414.861277<br>(hartrees)	gas phase en.: -414.284165<br>sol phase en.: -414.391998<br>(hartrees)
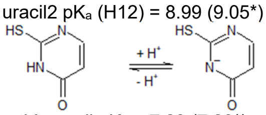<br>thiouracil pK_a = 7.28 (7.23*)	gas phase en.: -737.770685<br>sol phase en.: -737.789703<br>(hartrees)	gas phase en.: -737.223094<br>sol phase en.: -737.326648<br>(hartrees)
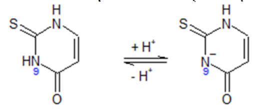<br>thiouracil2 pK_a (H9) = 7.14 (7.15*)	gas phase en.: -737.790006<br>sol phase en.: -737.814698<br>(hartrees)	gas phase en.: -737.814698<br>sol phase en.: -737.350178<br>(hartrees)
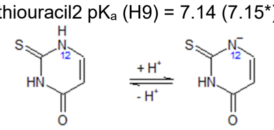<br>thiouracil2 pK_a (H12) = 6.53 (6.54*)	gas phase en.: -737.782590<br>sol phase en.: -737.240363<br>(hartrees)	gas phase en.: -737.804492<br>sol phase en.: -737.340535<br>(hartrees)
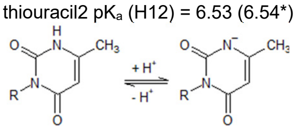<br>compound 15 pK_a = 8.81	gas phase en.: -647.324351<br>sol phase en.: -647.350320<br>(hartrees)	gas phase en.: -646.780623<br>sol phase en.: -646.880322<br>(hartrees)
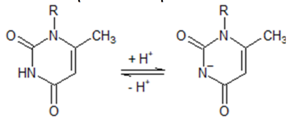<br>compound 18 pK_a = 8.13	gas phase en.: -608.005898<br>sol phase en.: -608.034294<br>(hartrees)	gas phase en.: -607.449338<br>sol phase en.: -607.565729<br>(hartrees)

** these values were obtained with the jaguar pKa genereated conformers*

Discussion

For the elucidation of the results obtained, theoretical calculations were carried out and pKa values were determined for compounds **1**–**7**. [32–34] In the case of **1**–**3**, there is only a single protic hydrogen in the molecules, attached to the nitrogen atom with calculated pKa values of 8.63, 9.70 and 11.89, respectively. These data indicate that under alkaline conditions, protons can be removed and the formed anions as nucleophiles are capable of opening PC providing the *N*-alkylated product. Considering compound **4**, there is only single NH in the molecule with a protic nature, but both nitrogen atoms can carry protic hydrogens. As a consequence, two products are expected. The pKa values calculated are 7.91 and 8.40 for 1*N* and 3*N*, respectively, indicating that hydrogen in the 1*N* position is more acidic. This corroborates our experimental results, since the 1*N*-alkylated product was formed in higher amount. Importantly, no doubly-alkylated product was observed. As regards compound **5**, each nitrogen atom bears a hydrogen of protic nature, thus double alkylation might be expected. The pKa values calculated are 9.44 and 9.34 for 1*N* and 3*N*, respectively. According to these data, the first alkylation may takes place in position 3. The pKa value calculated for the 3*N*-alkylated derivative of **5** is 8.81. Accordingly, the alkylation of **5** in position 3 enhanced the acidity of position 1 and this explains why the doubly-alkylated product was isolated as the sole derivative. Concerning compound **6**, there is only a single hydrogen of protic character in the molecule. However, the anion formed after deprotonation has two isomeric forms with the negative charge located at either in position 1 or position 2. Theoretical calculations suggest that the latter isomeric form is slightly more stable (~1 kcal/mol), but charge transition is possible, due to the low energy difference between the two isomeric forms. Finally, for compound **7**, results may be expected to be similar to those of **5**. The pKa values calculated are 6.54 and 7.15 for 1*N* and 3*N*, respectively. Both protons have significant acidic nature, that is, doubly alkylation observed is in harmony with theoretical calculations (Table S9).
